# Competing Mechanisms in Palladium-Catalyzed Alkoxycarbonylation of Styrene

**DOI:** 10.1021/acscatal.4c00966

**Published:** 2024-04-01

**Authors:** Jaya Mehara, Mariarosa Anania, Pavel Kočovský, Jana Roithová

**Affiliations:** † Department of Spectroscopy and Catalysis, Institute for Molecules and Materials, Radboud University Nijmegen, Heyendaalseweg 135, Nijmegen 6525 AJ, The Netherlands; ‡ Department of Organic Chemistry, Faculty of Science, Charles University, Hlavova 2030/8, Prague 2 12843, Czech Republic; § Institute of Organic Chemistry and Biochemistry, Czech Academy of Sciences, Flemingovo nám. 2, Prague 6 16610, Czech Republic

**Keywords:** palladium, copper, CO, mass spectrometry, reaction monitoring, reaction intermediate, isotopic labeling

## Abstract

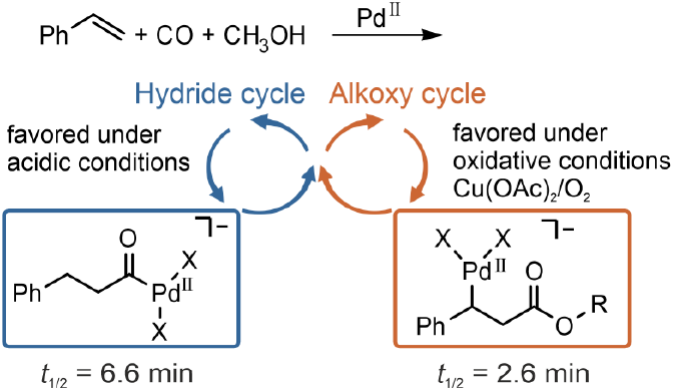

Palladium-catalyzed carbonylation is a versatile method for the synthesis of various aldehydes, esters, lactones, or lactams. Alkoxycarbonylation of alkenes with carbon monoxide and alcohol produces either saturated or unsaturated esters as a result of two distinct catalytic cycles. The existing literature presents an inconsistent account of the procedures favoring oxidative carbonylation products. In this study, we have monitored the intermediates featured in both catalytic cycles of the methoxycarbonylation of styrene PhCH=CH_2_ as a model substrate, including all short-lived intermediates, using mass spectrometry. Comparing the reaction kinetics of the intermediates in both cycles in the same reaction mixture shows that the reaction proceeding via alkoxy intermediate [Pd^II^]-OR, which gives rise to the unsaturated product PhCH=CHCO_2_Me, is faster. However, with an advancing reaction time, the gradually changing reaction conditions begin to favor the catalytic cycle dominated by palladium hydride [Pd^II^]-H and alkyl intermediates, affording the saturated products PhCH_2_CH_2_CO_2_Me and PhCH(CO_2_Me)CH_3_ preferentially. The role of the oxidant proved to be crucial: using *p*-benzoquinone results in a gradual decrease of the pH during the reaction, swaying the system from oxidative conditions toward the palladium hydride cycle. By contrast, copper(II) acetate as an oxidant guards the pH within the 5–7 range and facilitates the formation of the alkoxy palladium complex [Pd^II^]-OR, which favors the oxidative reaction producing PhCH=CHCO_2_Me with high selectivity. Hence, it is the oxidant, rather than the catalyst, that controls the reaction outcome by a mechanistic switch. Unraveling these principles broadens the scope for developing alkoxycarbonylation reactions and their application in organic synthesis.

## Introduction

**Scheme 1:**
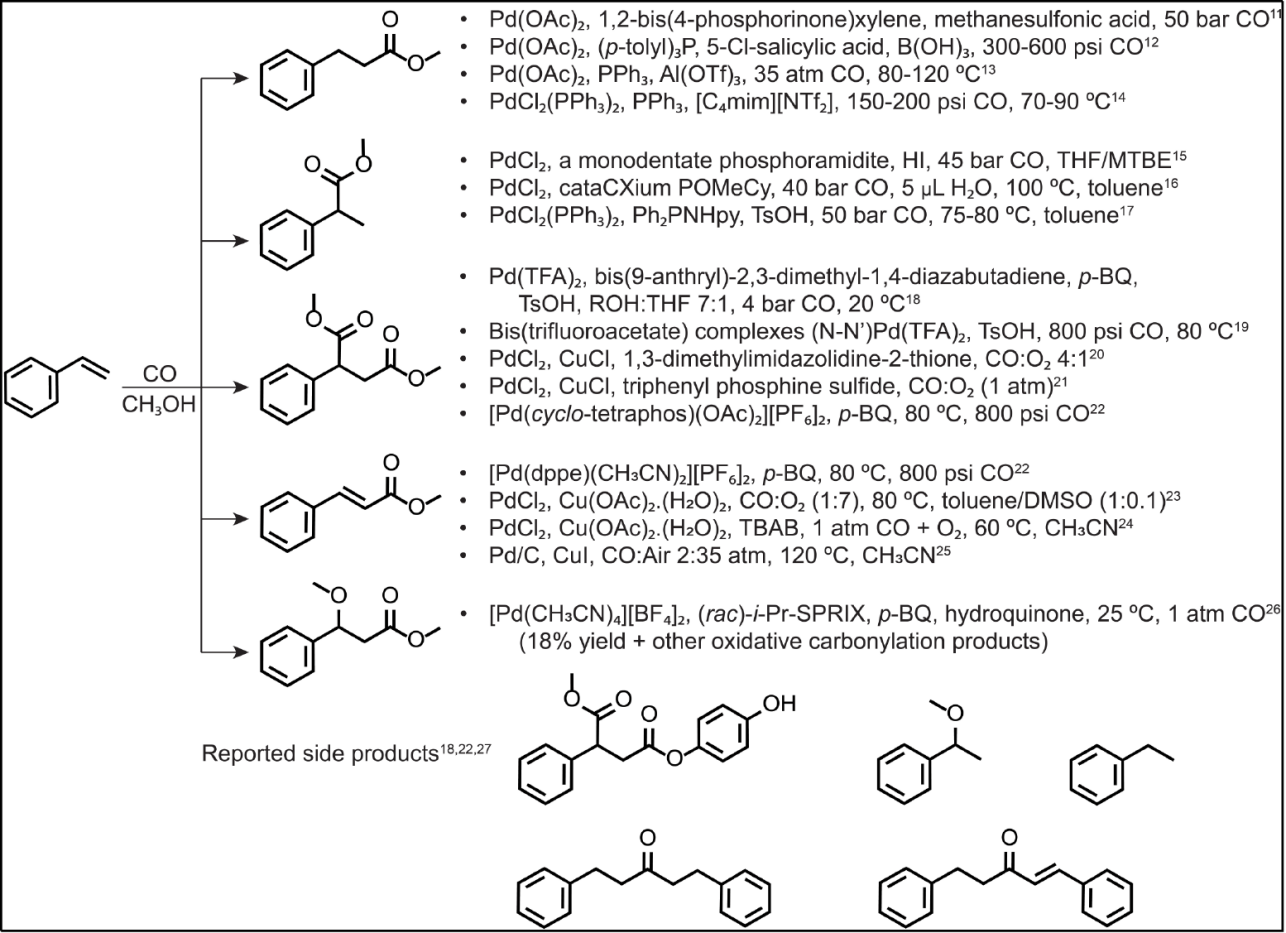
Different Conditions Steer the Methoxycarbonylation of Styrene to Different Products For more details on the ligands employed, please refer to the original literature.

Carbonylation reactions convert alkenes/alkynes into aldehydes, acids, esters, or lactones in a one-pot catalytic reaction.^1−3^ The catalysts are usually based on transition metals, such as Rh, Ru, Ir, Pd, Co, and Fe.^1,4^ Palladium-catalyzed carbonylation reactions, in particular, have garnered significant attention owing to their potentially broad applicability.^5,6^ However, unless an intervention of a neighboring group is involved,^7−10^ these reactions typically exhibit low selectivity; thus, carbonylation of α-olefins can give various products with only a limited level of control (Scheme 1), which reduces the synthetic applicability of this methodology. Nevertheless, empirical tuning of the reaction conditions by selection of the ligand (phosphines, imines, NHCs, thioureas, etc.), oxidant, additives (acids, salts), temperature, and CO pressure, has allowed, to some extent, to steer the reaction toward the desired product (Scheme 1).^11−27^

**Figure 1:**
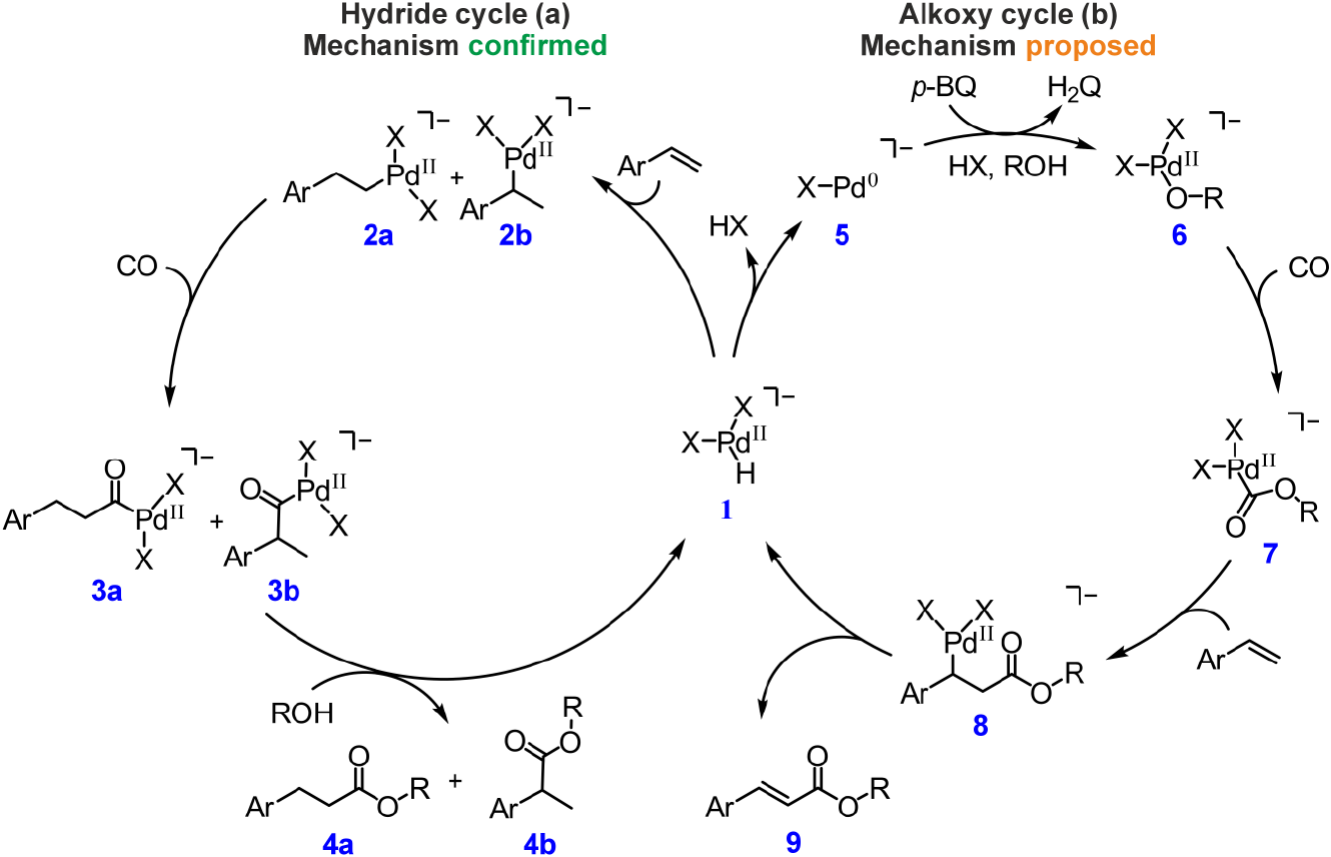
Palladium-catalyzed carbonylation: (a) palladium-hydride mechanism and (b) palladium-alkoxy mechanism. The formulas assume negatively charged ligands “X.” If one or both “X” are neutral ligands, then the overall charge of the complexes changes accordingly.

Previous mechanistic studies suggested that alkoxycarbonylation can occur via two competing pathways (Figure 1). The first pathway (“hydride cycle”) involves palladium(II) hydride complex [Pd^II^]-H (**1**) as the key intermediates. The palladium hydride complexes react with alkenes by 1,2-insertion to form the palladium alkyl complexes **2a**/**2b**. In the next step, a 1,1-insertion of CO leads to the formation of the acyl-palladium complexes **3a**/**3b**. The final step is alcoholysis, leading to the saturated ester product **4a**/**4b** and regeneration of palladium(II) hydride intermediate **1**.^28−30^ This “hydride” mechanism is favored under acidic conditions^31^ and does not require an additional oxidant.

All intermediates in the hydride cycle were studied by NMR spectroscopy.^32−38^ The structures of palladium hydride complexes and acyl-palladium complexes were characterized by X-ray spectroscopy.^33,38,39^ Further mechanistic studies involved DFT, deuterium labeling, HPLC-MS, and GC-MS techniques.^33,34,39−41^

The alternative mechanism (“alkoxy cycle”) can be conjectured to feature alkoxy-palladium complexes **6** as the key intermediates, which are likely to insert CO, generating alkoxycarbonyl palladium intermediates **7**. Subsequent steps would involve 1,2-insertion of an alkene (**8**), followed by β-H elimination, ultimately yielding unsaturated ester product **9** and palladium-hydride complex **1** in analogy with the classical Heck reaction (Figure 1). Palladium hydride **1** can release a proton (H^+^) or eliminate an acid (HX) by reductive elimination, leading to the formation of palladium(0) complex **5**. The alkoxy cycle would then continue with the reoxidation of palladium(0) concomitant with the reformation of alkoxy-palladium complex **6**. For phosphine-based ligands, alkoxycarbonyl palladium complexes were prepared and studied previously by NMR spectroscopy.^42,43^

Controlling the selectivity of the alkoxycarbonylation reaction would require controlling the competition between these two cycles at the branching point of the formation of palladium hydride **1**. The alkoxy cycle can be easily suppressed by adding an acid, which disfavors the reductive elimination step. The reaction then proceeds selectively toward the saturated products **4a**/**4b**.^16,36−38,41^ The conditions favoring unsaturated products are more challenging to achieve.^23,24^

Several protocols for selective alkoxycarbonylation to afford unsaturated products were published, which, however, do not offer common ground for a mechanistic rationale of the observed selectivity.^22−25,42,43^ One of us has previously highlighted the significant role of acetonitrile in the oxidative alkoxycarbonylation of terminal alkenes toward α,β-unsaturated esters, suggesting the key role of acetonitrile as a ligand to the palladium intermediates, provided Cu(OAc)_2_·H_2_O is employed as the oxidant.^24^ Nevertheless, the actual role of this ligand, i.e., how it swings the reaction toward the unsaturated ester, was unclear. Furthermore, when CuCl_2_ was used instead of Cu(OAc)_2_, the reaction did not proceed even in acetonitrile.^24^ Wang et al., who used copper(II) acetate, also reported a palladium-catalyzed oxidative alkoxycarbonylation of alkenes.^23^ Unlike the previous study, which emphasized the importance of acetonitrile as a ligand, Wang et al. employed a toluene/dimethyl sulfoxide (DMSO) mixture as a solvent with no additional ligands. They proposed that acetate ions play a key role in alcohol activation and Pd(II) regeneration. In this respect, it is pertinent to note that various reactions based on palladium catalysts and DMSO (or its congeners) as a ligand have been reported, where oxygen acts as the sole oxidant for Pd(II) regeneration.^44−51^ Maffei et al. utilized a heterogeneous palladium source (Pd/C) and copper iodide to achieve effective oxidative alkoxycarbonylation of olefins to produce unsaturated esters.^25^ They suggested that this process required homogeneous palladium (leaching from the support) and a low concentration of carbon monoxide. In the very first example of oxidative carbonylation, Cometti and Chiusoli demonstrated the conversion of styrene into methyl cinnamate and also noted that the low CO pressure favored the desired product.^52^ On the other hand, higher CO pressure, in combination with certain ligands, led to the formation of the dicarbonylated products.^25,52^ Bianchini et al. successfully controlled the selectivity of the methoxycarbonylation of styrene by steering it either toward methylcinnamate or to dimethyl phenyl succinate by employing palladium precursors with various phosphine ligands.^22^

In light of the numerous conditions published to date, we set out to decipher the competition between the two mechanisms. Using mass spectrometry for monitoring the reactive intermediates, we now investigated the effect of various reaction conditions on the reaction kinetics and the proportional occurrence of the individual pathways.

## Results and Discussion

We focused on the interplay of intermediates in the two catalytic cycles in the methoxycarbonylation of styrene (Figure 1) under oxidative conditions by electrospray ionization mass spectrometry (ESI-MS). This technique is particularly suited for detecting low-abundance intermediates present in reaction mixtures.^53−55^ We mainly focused on PdCl_2_ as the catalyst in an acetonitrile/methanol solvent mixture using *p*-benzoquinone or Cu(OAc)_2_·H_2_O as oxidants. In addition, we also tested toluene/DMSO solvent and PdCl_2_(PPh_3_)_2_ and PdCl_2_(dppf) catalysts. The intermediates were detected as negative ions in the absence of phosphine ligands (X = Cl^−^ in Figure 1) or as positive ions when supported by phosphines (both X are neutral phosphine ligands in Figure 1, changing the charge of the complexes to + 1; see Figures S1–S4 and S37–S45).

### The Palladium-Hydride Cycle

**Figure 2:**
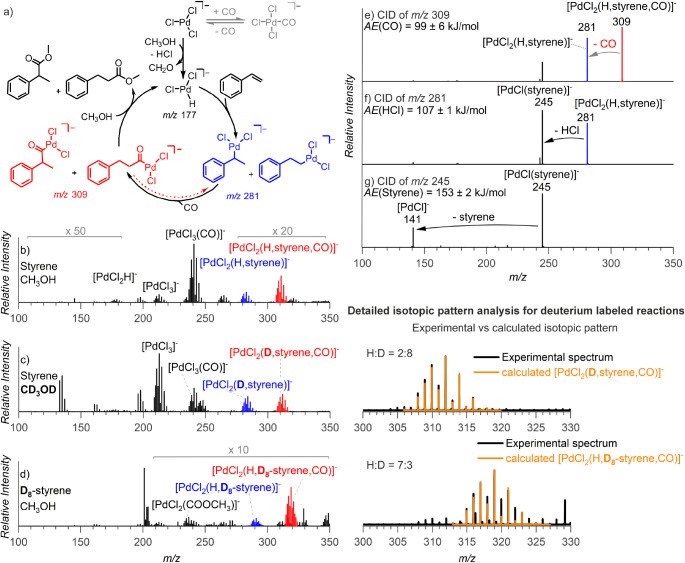
Palladium-hydride cycle: (a) Reaction mechanism with the detected intermediates. (b–d) Electrospray-ionization mass spectra of the reaction mixture; (b) PdCl_2_ (100 μM), *p*-benzoquinone (1 mM), styrene (5 mM) in CH_3_CN and CH_3_OH (1:1) under the CO atmosphere at 40 °C; (c) styrene with CD_3_OD; and (d) D_8_-styrene with CH_3_OH, the detailed spectra on the right show the isotopic patterns of the acyl-palladium complexes; the calculated H:D ratio refers to the hydrogen atom build-in as hydride; the ratio varied between 1: 9 and 3: 7 depending on the reaction time. (e–g) Collision-induced dissociation spectra of mass-selected ions indicated in the graphs. Appearance energies (*AE*s) were determined from energy-resolved experiments (see Figures S21–S23).

First, we monitored the reaction mixture of PdCl_2_, *p*-benzoquinone, styrene, and CO in CH_3_CN/CH_3_OH by offline sampling (for details, see Supporting Information). Aliquots of the reaction mixture were filtered, diluted, and analyzed by using ESI-MS (Figures 2b and S1–S3). Initially, we observed [PdCl_3_]^−^ and [PdCl_3_(CO)]^−^ as the dominant species. Other palladium complexes included the hydrido-palladium complex [PdCl_2_(H)]^−^ (*m*/*z* 177) and complexes formally corresponding to [PdCl_2_(H,styrene)]^−^ (*m*/*z* 281, blue) and [PdCl_2_(H,styrene,CO)]^−^ (*m*/*z* 309, red) (Figure 2b). These palladium complexes were stable at room temperature and could be detected over a long reaction time (20–120 min).

We further investigated the detected complexes by collision-induced dissociation (CID) experiments (Figure 2e–g). The detected [PdCl_2_(H,styrene)]^−^ intermediate, arising from [PdCl_2_(H,styrene,CO)]^−^ (Figure 2e), loses HCl (Figure 2f), indicating that it corresponds to the σ-bound alkyl palladium [PdCl_2_(CH_2_-CH_2_-Ph)]^−^ or [PdCl_2_(CH(CH_3_)-Ph)]^−^ rather than to a complex with π-coordinated styrene, which would be expected to lose preferentially styrene. The observed HCl loss must be preceded by the reverse 1,2-insertion of styrene (blue in Figure 2). Hence, the [PdCl(styrene)]^−^ fragment should contain π-coordinated styrene. Accordingly, [PdCl(styrene)]^−^ eliminates neutral styrene in the follow-up collision-induced dissociation experiment (Figure 2g).

The detected [PdCl_2_(H,styrene,CO)]^−^ complex eliminates CO (red in Figures 2b,e) in the collision-induced dissociation. The CO elimination can be interpreted either as the reversed 1,1-insertion reaction from [PdCl_2_(CO-C_2_H_4_Ph)]^−^ or as dissociation of CO from [PdCl_2_(C_2_H_4_Ph)(CO)]^−^. To assess which complex was detected, we compared the experimental appearance energies of the given fragmentations (*AE*s) with the theoretically calculated bond-dissociation energies (*BDE*s). The calculated *BDE* of CO in the [PdCl_2_(CH(CH_3_)-Ph)(CO)]^−^ and [PdCl_2_(CH_2_-CH_2_-Ph)(CO)]^−^ complexes, featuring tetra-coordinated palladium with one ligand being CO, is 71 kJ mol^–1^ and 77 kJ mol^–1^, respectively (Table S1). On the other hand, the *BDE* of CO in the acyl-palladium intermediates [PdCl_2_(CO-CH(CH_3_)-Ph)]^−^ and [PdCl_2_(CO-CH_2_-CH_2_-Ph)]^−^ are 95 kJ mol^–1^ and 96 kJ mol^–1^, respectively. Since the latter figures are consistent with the experimental value of 99 ± 6 kJ mol^–1^ obtained by the energy-resolved collision-induced dissociation experiment,^56,57^ the detected complex [PdCl_2_(H,styrene,CO)]^−^ can be assigned the acyl-palladium structure, i.e., [PdCl_2_(CO-C_2_H_4_-Ph)]^−^.

The [PdCl_2_(CO-C_2_H_4_-Ph)]^−^ assignment was corroborated by isotopic labeling: In analogy with the previous experiment, the reaction of D_8_-styrene and D_4_-methanol generated the perdeuterated complex [PdCl_2_(CO-C_2_D_4_-Ph^D5^)]^−^ (*m*/*z* 318, Figure S10). Collision-induced dissociation of this intermediate led to the consecutive elimination of the CO and DCl, as expected. The reaction of D_8_-styrene and unlabeled methanol led to [PdCl_2_(CO-C_2_D_3_H-Ph^D5^)]^−^ (*m*/*z* 317, Figure 2d), which sequentially extruded CO and DCl or HCl in the collision experiments (Figure S11).

Using ion-mobility separation (IMS), we endeavored to identify the isomers of the detected ions. Under catalytic conditions (*p*-benzoquinone as an oxidant), we observed only single IMS peaks for the detected palladium complexes (Figure S27). Interestingly, with a stoichiometric PdCl_2_ concentration, the acyl-palladium intermediates showed two isomers, indicating a reduced selectivity (Figures S28, S29, and Table S3). The appearing minor isomer has a smaller size (collision cross section) than the dominant isomer that prevails at the catalytic conditions. According to theoretical calculations, the branched [PdCl_2_(CO-CH(CH_3_)-Ph)]^−^ isomer is smaller (Table S3). Hence, the major detected acyl-palladium complexes correspond to linear isomer [PdCl_2_(CO-CH_2_-CH_2_-Ph)]^−^.

For the alkylpalladium complexes, we observed both isomers only if we used D_8_-styrene and a stoichiometric amount of PdCl_2_ (Figure S29). The minor isomer is larger than the dominant one (Figure S29). The calculations again suggest that the branched isomer is smaller than the linear isomer (Table S2). Accordingly, the dominant alkyl palladium complex detected from the solution is the branched isomer [PdCl_2_(CH(CH_3_)-Ph)]^−^.

The final confirmation of the structure for the observed complexes was obtained by using infrared multiphoton dissociation (IRMPD) spectroscopy. The IRMPD spectrum of mass-selected [PdCl_2_(H,styrene)]^−^ (*m*/*z* 281, Figure S30) agrees better with the theoretical IR spectrum of the branched alkylpalladium complex. The IRMPD spectrum of [PdCl_2_(H,styrene,CO)]^−^ (*m*/*z* 309, Figure S31) agrees best with that of the linear [PdCl_2_(CO-CH_2_-CH_2_-Ph)]^−^ complex.

The findings about the detected isomeric palladium complexes can serve as a nice demonstration of the Curtin–Hammett principle.^58^ The branched α-phenylalkyl palladium complexes are more stable than their linear counterpart β-phenylalkyl palladium complexes (Table S2). Therefore, they prevail in the solution. However, the linear alkyl palladium complexes react faster in the insertion step, which results in the predominant formation of the linear products (Table S3).

**Scheme 2:**
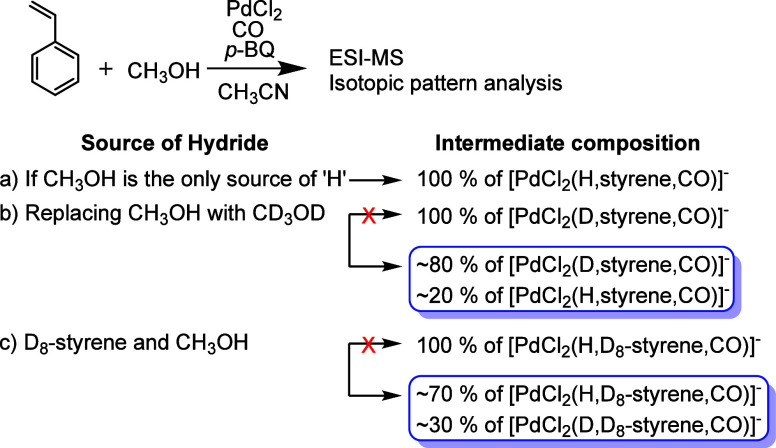
Summary of the Labeling Experiment and the Resulting Isotopic Composition of the Hydride Intermediate [PdCl_2_(H,styrene,CO)]^−^ Observed Experimentally (Figure 2c,d).

Finally, we addressed the origin of the hydride ligand in **1** (Figure 1). A priori, the palladium hydride intermediates **1** can either be formed by the reaction of the starting palladium complex with methanol or originate from β-hydrogen elimination of the styrene-containing intermediate **8** in the alkoxy-cycle (Figure 1). To shed light on this issue, we further analyzed the labeling experiments. In the reaction of unlabeled styrene with CD_3_OD (Figure 2c, Scheme 2b), the ESI-MS spectra showed an almost exclusive formation of [PdCl_2_(D,styrene,CO)]^−^ (*m*/*z* 310), which suggests that the hydride originates from methanol. However, the ion intensities in the isotopic pattern do not match exactly, indicating the presence of about 20% [PdCl_2_(H,styrene,CO)]^−^ (*m*/*z* 309) with H originating from β-hydrogen elimination from the styrene-containing intermediates. In the reaction of labeled D_8_-styrene with unlabeled CH_3_OH (Figure 2d), we detected [PdCl_2_(H,D_8_-styrene,CO)]^−^ (*m*/*z* 317). Closer inspection of the isotopic pattern revealed about 30% contribution of [PdCl_2_(D,D_8_-styrene,CO)]^−^ (*m*/*z* 318) with D arising by β-deuterium elimination from the D_8_-styrene-containing intermediate. These results demonstrate the interconnection of both cycles via a hydride intermediate.

### The Alkoxy-Palladium Cycle

The operation of this cycle was inferred from the generally observed reactivity of the palladium complexes. However, to date, the intermediates have not been detected directly. Since we assumed them to be short-lived, we opted for the pressurized sample infusion-electrospray ionization-mass spectrometry(PSI-ESI-MS) monitoring.^59,60^ Our experiments showed that indeed the intermediates can be detected at room temperature using a CO balloon at reaction times below 30 min. At longer times, the palladium hydride intermediates prevailed (Figure S34). The onset of the palladium-hydride intermediates is linked with the occurrence of palladium black in the reaction vessel, suggesting an insufficient regeneration of the palladium(II) complex, which then results in the formation of larger palladium(0) clusters.

**Figure 3:**
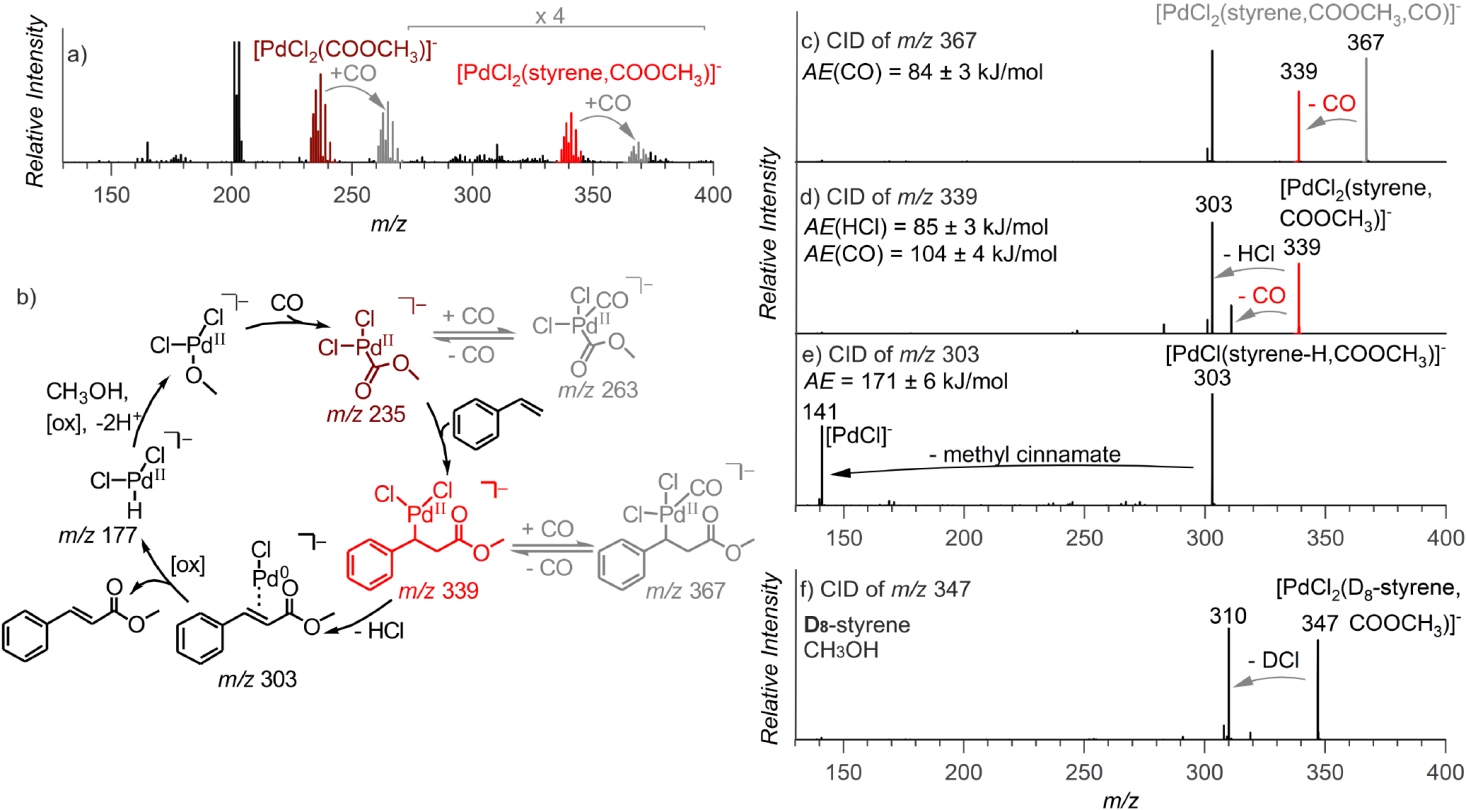
Palladium-alkoxy cycle: (a) PSI-ESI-MS spectrum of the reaction mixture of PdCl_2_ (100 μM), *p*-benzoquinone (1 mM), and styrene (5 mM) in acetonitrile and CH_3_OH (1:1) ratio under the CO atmosphere (using a balloon) monitored online for < 20 min at room temperature; (b) reaction mechanism with the detected intermediates; (c–f) collision-induced dissociation spectra of the indicated mass-selected ions. Appearance energies (*AE*s) were determined from the energy-resolved collision-induced dissociation experiments (see Figures S24–S26).

At short reaction times, we have detected the Pd-methoxycarbonyl precursor complexes [PdCl_2_(COOCH_3_)]^−^ (*m/*z 235) and [PdCl_2_(COOCH_3_,CO)]^−^ (*m*/*z* 263) (Figure 3a,b); this assignment was confirmed by the experiments carried out in CD_3_OD (Figures 3 and S1–S4). The initially formed [PdCl_2_(COOCH_3_)]^−^ complex undergoes fragmentation by the elimination of α-acetolactone (Figure S5) to give the [Pd^II^Cl_2_(H)]^−^ fragment. Similarly, [PdCl_2_(COOCD**_3_**)]^−^ (*m/*z 238) eliminates deuterated α-acetolactone and yields [Pd^II^Cl_2_(D)]^−^. Interestingly, the kinetic isotope effect in the deuterated intermediate decreases the abundance of the rearrangement required for this fragmentation and opens the competing fragmentation path via the loss of CO (Figure S6). The Pd-methoxycarbonyl intermediates were also observed to arise in toluene and other nonpolar solvents or in the presence of phosphine ligands (Figures S39 and S40).

After the addition of styrene to the reaction mixture containing the alkoxy precursor complexes, the formation of styrene-containing intermediates [PdCl_2_(styrene,COOCH_3_)]^−^ (*m*/*z* 339) was observed (Figure 3b). Notably, the latter intermediate adds another CO molecule to the fourth coordination site, yielding [PdCl_2_(styrene,COOCH_3_,CO)]^−^ (*m*/*z* 367) (Figure 3b). In collision-induced dissociation, the [PdCl_2_(styrene,COOCH_3_)]^−^ complex lost HCl (Figure 3d), which corresponds to β-hydrogen elimination. The fragmentation appeared at a low collision energy (85 ± 3 kJ mol^–1^), indicating a low energy barrier for this process.

### Interplay of the Catalytic Cycles

The decisive splitting between the hydride and alkoxy catalytic cycles occurs at the point of palladium hydride formation (Figure 1). Either the hydride inserts the alkene (**1 → 2a/2b**) or is transformed into the alkoxypalladium complex (**1** → **6**). Hence, the kinetics of these steps affect the selectivity of the reactions. To shed light on the kinetics of these catalytic cycles, we employed delayed reactant labeling.^61,62^ This method allows us to study the half-lives of the detected intermediates in solution by monitoring the equilibration of their isotopologs. Here, we added D_5_-styrene to the running reaction mixture of unlabeled reactants. In time, we observed the building up of the deuterated intermediates next to their unlabeled congeners already present. The kinetics of their equilibration reflect their lifetime in the solution.

**Figure 4:**
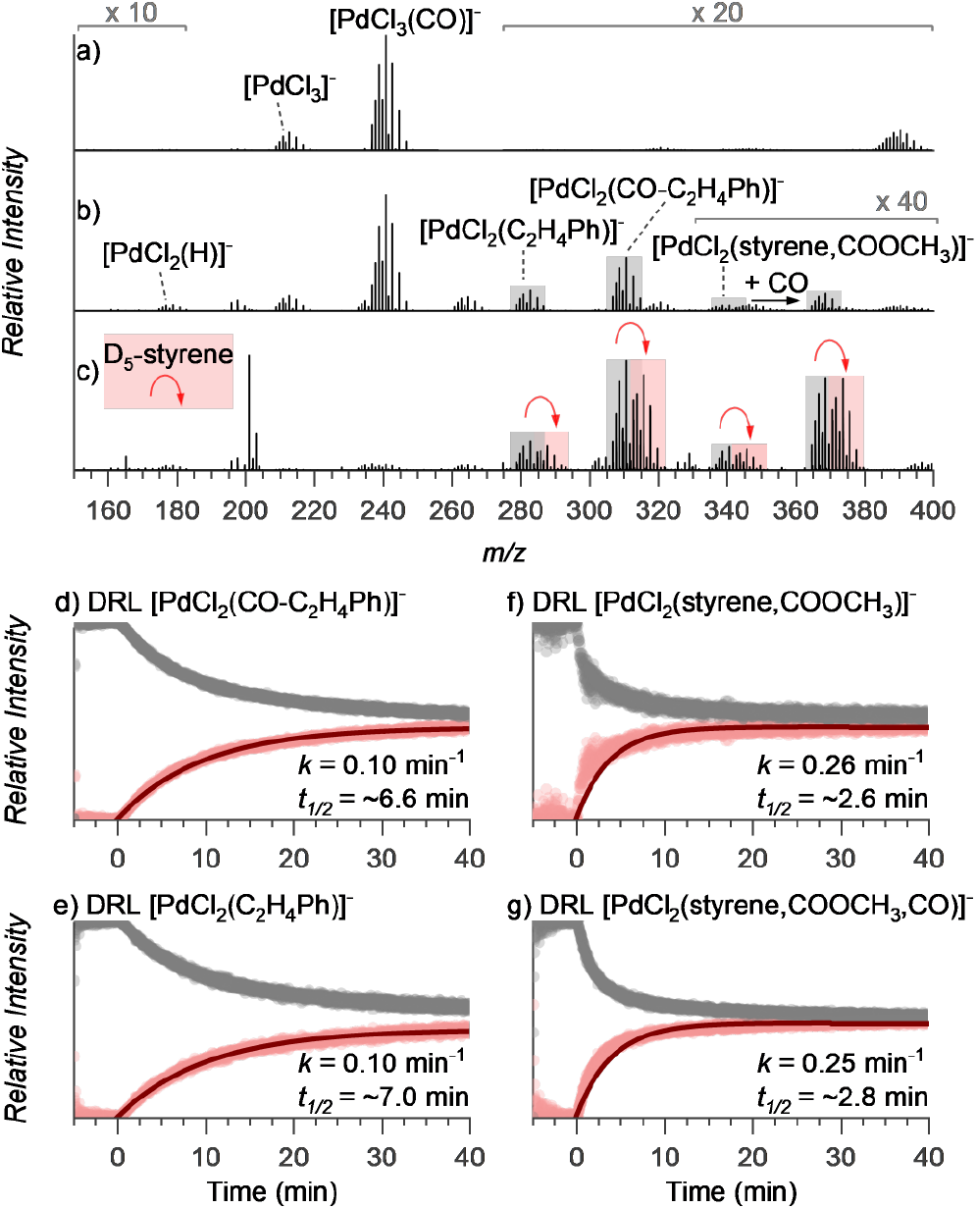
Snapshots of ESI-MS spectra during delayed reactant labeling experiments. The compositions were as follows: (a) PdCl_2_ and oxidant (*p*-benzoquinone) in acetonitrile prestirred under CO atmosphere at 40 °C for 10–20 min; (b) styrene in methanol was added; (c) after a delay of 5 min, D_5_-styrene in methanol was added and the reaction was further monitored by ESI-MS (red curly arrows indicate the corresponding intermediate with D_5_-styrene); (d–g) relative evolution of the signal intensities of the unlabeled (light gray) and labeled (light red) intermediates for the indicated complex. The fits (dark red) correspond to the *I*_t_ = *I*_eq_(1 – *e*^–*k*t^) function, where *I*_t_ and *I*_eq_ are relative signal intensities of the labeled ion and unlabeled ion at time *t* and the steady-state equilibrium, respectively, and *k* is the rate constant, *t*_1/2_ = ln2/*k*.

Figure 4 shows the experiment under the settings where the intermediates in both catalytic cycles are present in the reaction mixture. First, we set up the carbonylation reaction with unlabeled reactants. We detected the palladium hydride intermediates [PdCl_2_(C_2_H_4_Ph)]^−^ and [PdCl_2_(CO-C_2_H_4_Ph)]^−^ (Figure 4a,b), as well as the palladium alkoxy intermediates [PdCl_2_(styrene,COOCH_3_)]^−^ and [PdCl_2_(styrene,COOCH_3_,CO)]^−^. After 5 min, we added D_5_-styrene and saw the onset of the signals of the deuterated analogs of all detected intermediates (Figure 4c). The relative evolution of the nondeuterated and deuterated signals can be quantitatively evaluated because the ESI-MS detection efficiency is equal for different isotopologs (see Figure 4d–g).^63^ Accordingly, we fitted the isotopolog equilibration kinetics with an exponential fit (datapoints: light gray and red; fit: dark red lines in Figure 4d–g). The derived half-lifetimes of the intermediates in the solution revealed that the hydride-cycle intermediates have a much longer half-life (∼6.6 min) than the alkoxy-cycle intermediates (∼2.6 min).^60^ The half-lives of the hydride-cycle intermediates [PdCl_2_(C_2_H_4_Ph)]^−^ and [PdCl_2_(CO-C_2_H_4_Ph)]^−^ are comparable, demonstrating that the CO insertion is fast and does not represent a bottleneck of the reaction (compare Figure 4d,e). Hence, final methanolysis is most likely the rate-determining step in the hydride cycle.

The observed short lifetime of the intermediate [PdCl_2_(styrene,COOCH_3_)]^−^ in the alkoxy cycle suggests rapid β-hydrogen elimination, which agrees well with the low activation energy determined for the elimination of HCl from the gaseous complex (Figure 3d). Equilibration of the off-cycle intermediates [PdCl_2_(styrene,COOCH_3_,CO)]^−^ is slightly slower, suggesting that both of these palladium complexes are in equilibrium and that CO addition does not significantly affect the reaction (at the given pressure). We also tested the kinetics of equilibration of the Pd alkoxy precursor complex [PdCl_2_(COOCH_3_)]^−^ (*m/*z 235) with labeled CD_3_OH (Figure S32). As expected, these intermediates equilibrated almost immediately. Hence, the results clearly show that under the conditions that favor the formation of alkoxy intermediates, the catalytic cycle toward unsaturated products is faster and should, therefore, prevail. However, as the reaction progresses, the conditions in the reaction mixture begin to disfavor the formation of the alkoxy intermediates (vide infra), and, as a result, the hydride catalytic cycle starts to dominate.

To support the conclusion derived from the monitoring of the reactive intermediates, we also monitored the progress and selectivity of the methoxycarbonylation of styrene by gas chromatography (Figure S35a). Using a stoichiometric amount of *p*-benzoquinone and 25 mol % PdCl_2_ catalyst, we could detect about a 1: 1: 1 ratio of two saturated- and one unsaturated esters after 30 min of the reaction time. At longer reaction times, the saturated products started to prevail, and the unsaturated product was fully converted into the product of double methoxycarbonylation. We also tested the reaction under the same conditions but with a Cu(OAc)_2_ oxidant. The reaction was fully selective for the formation of the unsaturated ester product with a partial conversion to the product of double methoxycarbonylation (Figures S35b and S36).

### Steering the Catalytic Cycles

Understanding the differences between the two catalytic cycles opens the way to the identification of a possible steering wheel. As previously found, the pH of the reaction mixture is an important factor: In the presence of an acid, the hydride cycle becomes dominant, leading to an improved selectivity in carbonylation procedures by promoting the formation of the saturated products **4a/4b**. However, even without added acid, we observed significant variations in pH in experiments with various oxidants. Reaction mixtures with stoichiometric PdCl_2_ and *p*-benzoquinone became acidic (pH < 3) after 30 min, while those with copper acetate monohydrate as an oxidant retained the pH in the approximate range of 5–7. As demonstrated before, lower pH favors the hydride catalytic cycle.^16,17,29,37^ Hence, the choice of the oxidant is critical for the outcome of the carbonylation reactions.

**Figure 5:**
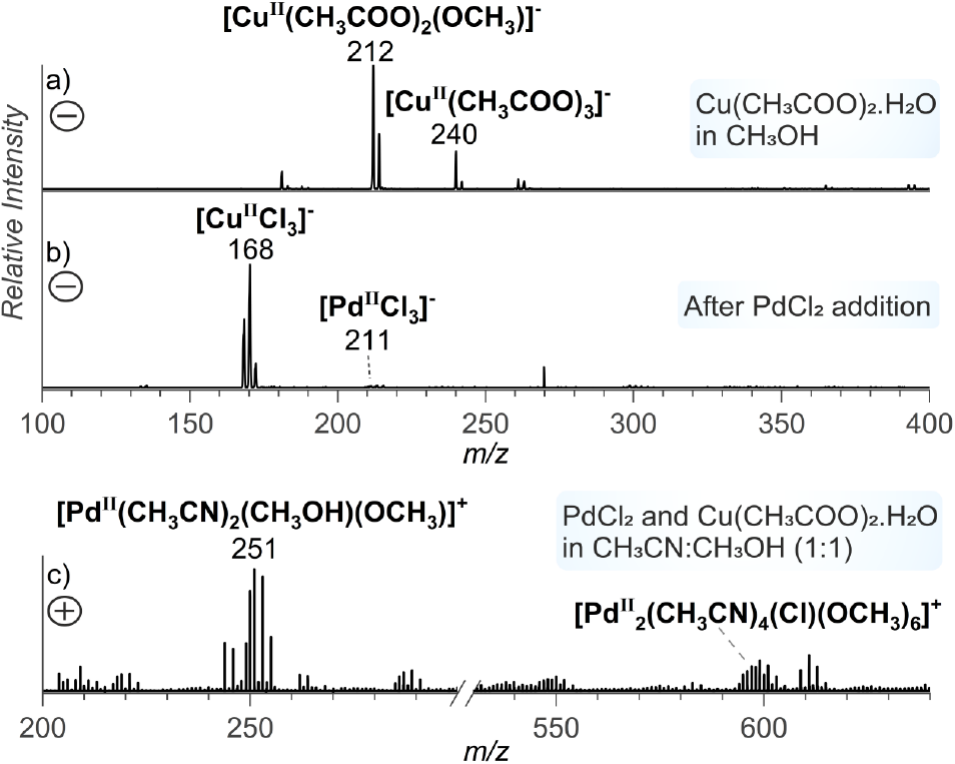
(a) ESI-MS spectrum (negative mode) of copper acetate monohydrate dissolved in methanol; (b) addition of PdCl_2_ to the solution of copper acetate monohydrate in methanol; (c) ESI-MS (positive mode) spectrum of copper acetate monohydrate and PdCl_2_ in a mixture of CH_3_CN:CH_3_OH.

The transformation of palladium(II) hydride **1** into the palladium(II)-alkoxy intermediate **6** is the key factor in steering the reactivity toward the unsaturated product **9**. To investigate the role of copper beyond being an oxidant in the catalytic cycle, we investigated the speciation of copper and palladium complexes in solution. Analysis of a solution of copper acetate in methanol revealed the presence of [Cu(OAc)_2_(OCH_3_)]^−^*m*/*z* 212 (Figure 5a), indicating the formation of methoxy complexes. Upon adding PdCl_2_ to the solution, the copper complexes are transformed into[CuCl_3_]^−^ ions (Figure 5b), suggesting the copper participation in facilitating the transfer of the methoxo and acetato ligands to the palladium center.^24,64^ The formation of mono- and di-Pd complexes with methoxy ligands is detected in positive ionization mode (Figures 5c, S46).

**Scheme 3:**
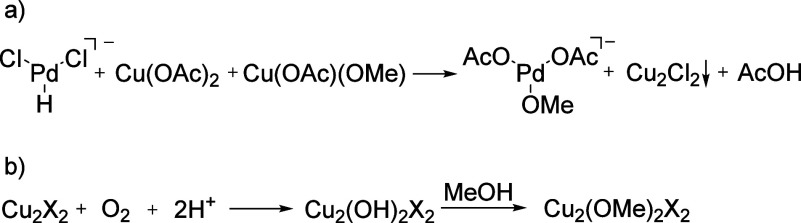
Reaction pathway for transforming [Pd]-H into [Pd]-OMe in the presence of copper acetate as an oxidant, based on the ions observed in Figure 5. “X” stands for any counter ion present in the solution.

**Figure 6:**
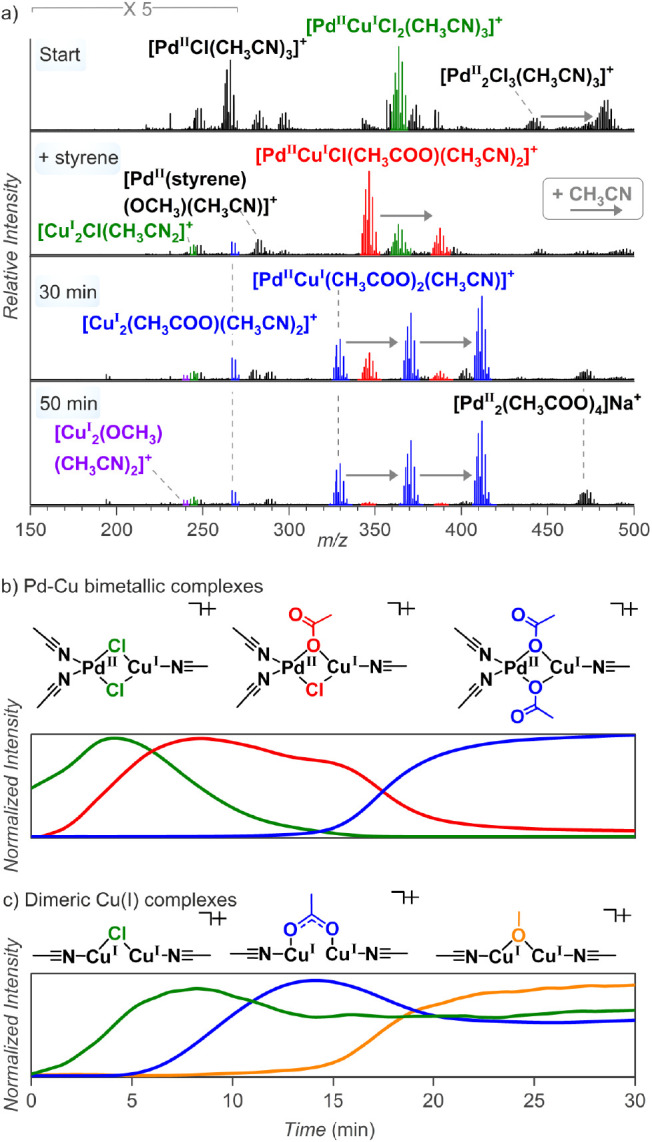
(a) Snapshots of the ESI-MS (positive mode) spectra of PdCl_2_ and copper acetate monohydrate in CH_3_CN:CH_3_OH under the CO and O_2_ atmosphere at room temperature: at the start, after styrene addition, 30 min, and 50 min; the gray arrows indicate the complex with additional CH_3_CN. (b) Plot of normalized traces of specified bimetallic complexes vs time; and (c) plot of normalized traces of specified dimeric Cu(I) complexes vs time. For more information, see Figure S48.

During the methoxycarbonylation reaction, we detected many mixed palladium-copper complexes, all corresponding to a palladium(II) interaction with copper(I). These complexes are thus likely intermediates in the reoxidation of palladium(0) by copper(II) in the solution. The speciation of the complexes changes with the progressing reaction time (Figure 6a). At the onset of the reaction, the complexes mostly contained Cl^−^ counterions. With increasing reaction time, Cl^–^ was progressively exchanged by AcO^–^ (Figure 6b, green → red → blue). Finally, we detected dimeric copper(I) complexes bridged by MeO^–^ (Figure 6c, orange). This observation is consistent with the removal of Cl^–^ by Cu^I^ from the solution (Scheme 3a). In addition, the reoxidation of copper(I) requires protons, which leads transiently to the formation of copper(II) hydroxide (Scheme 3b). This reaction helps to guard the pH during the reaction and supports the effective formation of the methanolato ligands required for the methoxycarbonylation reaction. Overall, this finding explains the significance of the copper-based oxidants in the alkoxy pathway, leading to the dominant formation of unsaturated esters (**9**) as the major products.

Our experiments were performed with the PdCl_2_ catalyst precursor. The results imply that using Pd(OAc)_2_ instead should be advantageous, as it would avoid the initial Cl^–^ to AcO^–^ exchange. The effect of Cl^–^ on pH using the copper oxidant is diminished due to Cu_2_Cl_2_ precipitation. However, when using *p*-BQ or other oxidants, a palladium salt with counterions from weaker conjugated acids (e.g., AcO^–^) should be more inclined to drive the reaction toward the alkoxy cycle, which agrees with our earlier findings.^24^

The mechanism of reoxidation of palladium(0) complexes with quinones was studied previously, and the η^2^-Pd(II)-(*p*-BQ) and η^2^-Pd(0)-(*p*-BQ) were intercepted by NMR spectroscopy.^65−68^ The reduction of *p*-benzoquinone to hydroquinone is associated with 2e^–^ oxidation of palladium(0) and consumption of 2 protons. Hence, the efficient oxidation of palladium(0) with *p*-benzoquinone should not lead to a dramatic pH drop. Nevertheless, hydroquinone is a weak acid (p*K*_a_ ∼ 10); hence, even if the oxidation were efficient, hydroquinone could contribute to the pH drop. Overall, the pH drop and low selectivity suggest that *p*-benzoquinone is not a good oxidant for oxidative palladium-catalyzed methoxycarbonylation reactions with PdCl_2_. However, under optimized conditions, using phosphine ligands to stabilize palladium complexes, quinone oxidants can be sufficient.^22^ Note, that in other reactions, not involving palladium-hydride intermediates, *p*-benzoquinone can serve as an excellent oxidant. For example, in 1,4-difunctionalization of 1,3-dienes and in allylic oxidation of olefins, both in the presence of acetic acid.^69−72^ However, these examples are mechanistically unrelated to the reaction studied here.

## Conclusions

Two competing catalytic cycles in the palladium-catalyzed methoxycarbonylation reaction of styrene were investigated by using electrospray ionization mass spectrometry (Figure 1): a catalytic cycle via alkylpalladium intermediates **2a**/**2b**, yielding the saturated products **4a**/**4b**, and a catalytic cycle via alkoxypalladium intermediates **6**, affording the unsaturated products **9**. All palladium intermediates in the negative charge state in the suggested catalytic cycles (Figure 1) were detected, including the short-lived, low-abundance alkoxycarbonyl species **8**, which had never been detected before.

Isotopic labeling (with deuterium) allowed us to study the kinetics of the reactions of the intermediates in both catalytic cycles simultaneously in the same reaction mixture. A direct comparison shows the following: The reaction proceeding via alkoxy intermediates, giving rise to the unsaturated product PhCH=CHCO_2_CH_3_, is faster than the competing reaction occurring via the alkylpalladium intermediate from which the saturated isomeric products PhCH_2_CH_2_CO_2_CH_3_ and PhCH(CO_2_CH_3_)CH_3_ are obtained. However, the conditions during the reaction evolve, manifested by a gradual suppression of the alkoxy palladium cycle and the ultimate domination of the palladium hydride and alkylpalladium intermediates, resulting in the formation of saturated products.

The oxidant has the key steering role in the reaction: *p*-benzoquinone causes a gradual decrease in pH, favoring the formation of palladium hydride intermediates, which leads to low reaction selectivity. By contrast, copper(II) acetate guards the pH in the 5–7 range. In addition, the copper ions form methoxy complexes by anion exchange with methanol, which facilitates the formation of palladium methoxy complexes. Hence, a mixture of copper(II) acetate and a palladium(II) salt is ideal for steering the alkoxy carbonylation reaction toward the unsaturated products, as demonstrated in the successful protocols—albeit until today without understanding the key role of the copper(II) acetate additive.

Overall, our findings provide the mechanistic understanding of the Pd-catalyzed alkoxycarbonylation of olefins but are likely to have significant implications in catalytic redox processes, far beyond the realm of palladium.

## Supporting Information Available

Experimental details and methods, ESI-MS and ESI-MS/MS, ion mobility analysis, IRMPD spectra, DRL experiments, DFT calculations (PDF)
