# Supplementary material for: Competing Mechanisms in Palladium-Catalyzed Alkoxycarbonylation of Styrene
Source: ACS Catal. 2024 Apr 1;14(8):5710–9. doi: 10.1021/acscatal.4c00966 (PMC11036401; doi:10.1021/acscatal.4c00966)
Supplement: Supplementary file 1 — cs4c00966_si_001.pdf [file cs4c00966_si_001.pdf]

Supporting Information

Competing Mechanisms in Palladium-Catalyzed Alkoxycarbonylation of Styrene

Jaya Mehara,[†] Mariarosa Anania,[†,‡] Pavel Kočovský,[‡,§] and Jana Roithová*[†]

[†] Department of Spectroscopy and Catalysis, Institute for Molecules and Materials, Radboud University Nijmegen, Heyendaalse-weg 135 6525 AJ Nijmegen (The Netherlands), E-mail: J.Roithova@science.ru.nl

[‡] Department of Organic Chemistry, Faculty of Science, Charles University, Hlavova 2030/8, 12843 Prague 2 (Czech Republic)

[§] Institute of Organic Chemistry and Biochemistry, Czech Academy of Sciences, Flemingovo nám. 2, 16610 Prague 6 (Czech Republic)

Table of Contents

Experimental details 3

Source spectra 5

The collision induced dissociation (CID) spectra of the ions with specified *m/z* 9

Energy Resolved Collision Induced Dissociation (CID) experiments 12

- Bond dissociation energy (*BDE*) calculation for CO loss from $[PdCl_2(H,styrene,CO)]^-$ *m/z* 309 16

Ion mobility separation 17

- Oxidant: *p*-benzoquinone (*p*-BQ) 17
- No oxidant, stoichiometric $PdCl_2$ with styrene 19
- No oxidant, stoichiometric $PdCl_2$ with D_8-styrene 20

Collision cross section (CCS) calculation 21

Delayed reactant labeling experiments 26

Effect of the reaction conditions on the observation of the intermediates 30

- 1,1′-Bis(diphenylphosphino)ferrocene (dppf) as the bidendate phosphine ligand 30
- Toluene/DMSO as the solvent instead of acetonitrile 31
- Toluene/DMSO as the solvent instead of acetonitrile and in the presence of monodentate triphenylphosphine ligand 31
- Standard reaction with *p*-benzoquinone as oxidant, acetonitrile and methanol as solvent under CO, but styrene substituted to *p*-chloro styrene 31
- Copper acetate monohydrate as oxidant and in the presence of tertabutyl ammonium bromide 32
- Palladium and copper speciation under the reaction conditions 34

xyz coordinates for optimized geometries 38

References 44

Experimental details

Mass spectrometric experiments were performed on Thermo Scientific LTQ XL, Finnigan LCQ Deca XP mass spectrometer, or Bruker TIMSTOF, all equipped with electrospray ionization (ESI) sources.[1,2] General conditions for LTQ and LCQ were as follows: sheath gas 5-40 arbitrary unit, auxiliary gas 0-10 arbitrary unit, capillary temperature 180-220 ℃, spray voltage 3-5 kV, capillary voltage -20 to 30 V, and tube lens -40 to 50 V. The energy-resolved collision-induced dissociation (CID) experiments were performed on LCQ Deca mass spectrometer with an ESI source. The negative mode collision energies in the LCQ ion trap were calibrated based on the measurements of dissociation energies of carboxylate anions (trifluoroacetate, dichloroacetate, trichloroacetate and benzoate).[3,4] The mass selected ions were collided with the He buffer gas with an activation time of 30 ms and activation q = 0.25. The complexes were repeated 2-4 times to determine the standard deviation of determined appearance energies (*AE*s).

General procedure for the offline sampling of reaction mixture:

To a solution of $PdCl_2$ (100 µM) in a mixture of acetonitrile (1 ml) and methanol (1 ml), *p*-benzoquinone (1 mM) was added. The clear solution was pre-stirred under the carbon monoxide (CO) atmosphere (using a balloon) for 2-5 minutes, and styrene (1-5 mM) was added. The reaction mixture was then stirred at 40 ℃ for 20-80 minutes. Aliquots of the reaction mixture were then filtered (using syringe filter) and analyzed on ESI-MS. The substrate and/or solvent were substituted for labeling experiments as indicated.

General procedure for the online sampling of reaction mixture i.e. pressurized sample infusion-electrospray ionization-mass spectrometry (PSI-ESI-MS)[5,6] *monitoring:*

To a solution of $PdCl_2$(100 µM) in a mixture of acetonitrile (1 ml) and methanol (1 ml), *p*-benzoquinone (1 mM) was added. The clear solution was pre-stirred under the carbon monoxide (CO) atmosphere (using a balloon) for 2-5 minutes. This stirred reaction mixture was directly sprayed into the ESI-MS inlet via silica capillary and N_2 or CO overpressure. When required using a heating metal block, the desired temperature was achieved. Substrate styrene (1-5 mM) was injected during the acquiring MS data to monitor the evolution of the reaction intermediates. The substrate and/or solvent were modified for the labeling experiments as indicated.

GC-TOF measurements: We performed 1:10 dilution of the reaction mixture at specified intervals with dichrolomethane (DCM), followed by extraction with water, resulting organic layer was dried over sodium sulphate, filtered by syringe filter and injected to the GC-TOF. The Agilent 7890A GC-TOF used was fitted with electron ionization (EI) source and HP-5MS column (30m x 0.25mm x 0.25µm). Method employed (15 min run time) started with the oven temperature at 100 ℃ (1 min hold), 20 ℃ increment until 320 ℃ (3 min hold). Detector was set at 2050 V and split ratio of 10.0:1 was applied.

pH measurements: As the reaction solvent(/s) i.e. acetonitrile and methanol are miscible with water, we prepared 1:1 dilution of the reaction mixture at selected interval with Milli-Q water, followed by mixing and syringe filtration to remove black precipitate. The resulting solution was tested with universal pH paper for qualitative pH estimation.

Source spectra

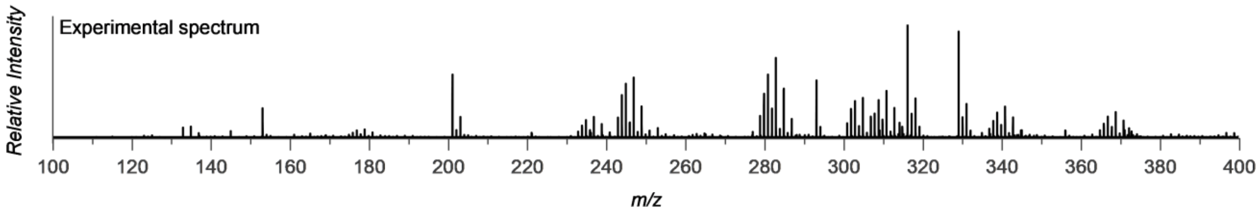

Figure S1: ESI-TOF spectra of the reaction mixture of $PdCl_2$(100 µM), *p*-benzoquinone (1 mM), styrene (5 mM) in acetonitrile and CH_3OH (1:1) ratio under CO atmosphere at 40 ℃ after 30 minutes, the black particles were filtered using syringe filter.

Below are the zoomed experimental spectrum sections (top) plotted against the calculated spectrum (bottom) for isotopic pattern analysis of the palladium complexes.

$[PdCl_2(H)]^-$ *m/z* 177

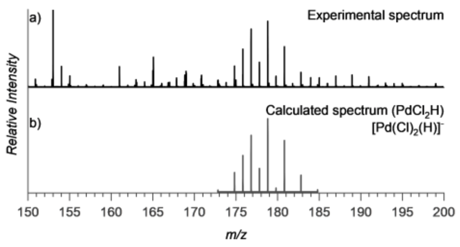

$[PdCl_2(COOCH_3)]^-$ *m/z* 235

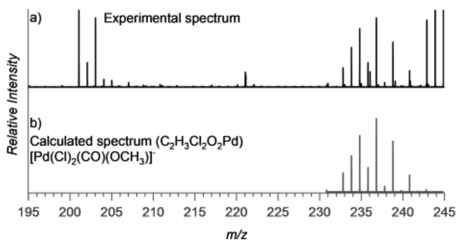

$[PdCl(styrene)]^-$ *m/z* 245

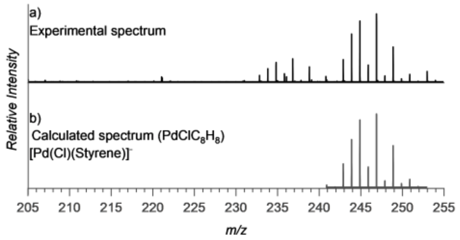

$[PdCl_2(styrene,COOCH_3)]^-$ *m/z* 339

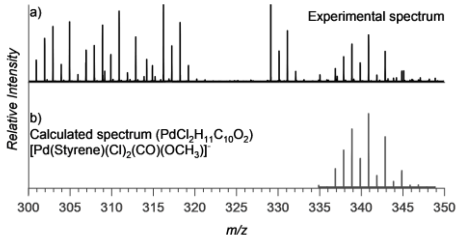

$[PdCl_2(H,styrene)]^-$ *m/z* 281

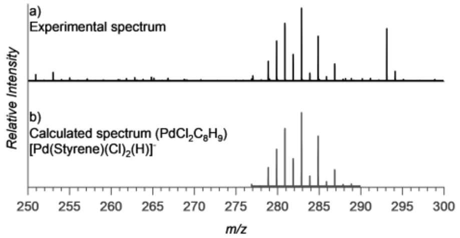

$[PdCl_2(styrene,COOCH_3,CO)]^-$ *m/z* 367

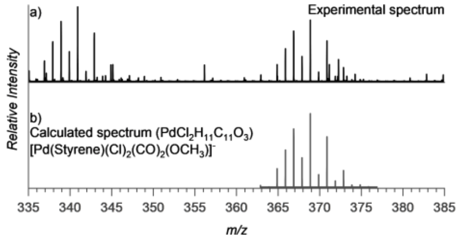

$[PdCl_2(H,styrene,CO)]^-$ *m/z* 309

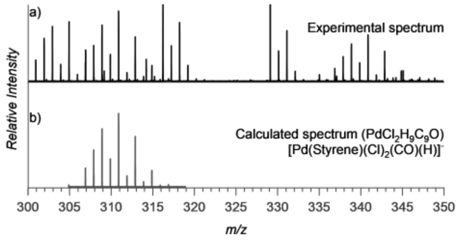

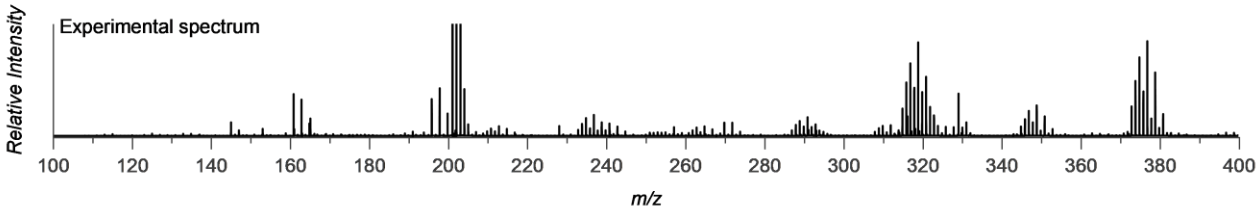

Figure S2: ESI-TOF spectra of the reaction mixture of $PdCl_2$(100 µM), *p*-benzoquinone (1 mM), D_8-styrene (5 mM) in acetonitrile and CH_3OH (1:1) ratio under CO atmosphere at 40 ℃ after 30 minutes, the black particles were filtered using syringe filter.

Below are the zoomed experimental spectrum sections (top) plotted against the calculated spectrum (bottom) for isotopic pattern analysis of the palladium complexes.

$[PdCl_2(H,D_8\text{-styrene},CO)]^-$ *m/z* 317

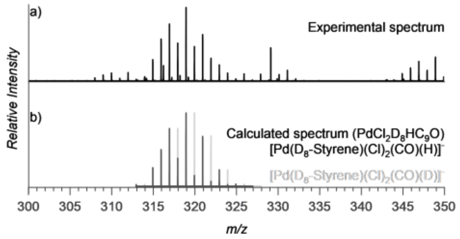

$[PdCl_2(D_8\text{-styrene},COOCH_3)]^-$ *m/z* 347

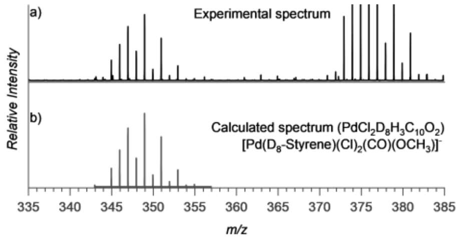

$[PdCl_2(D_8\text{-styrene},COOCH_3,CO)]^-$ *m/z* 375

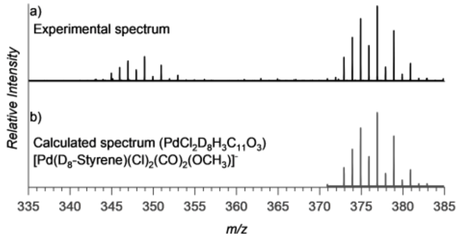

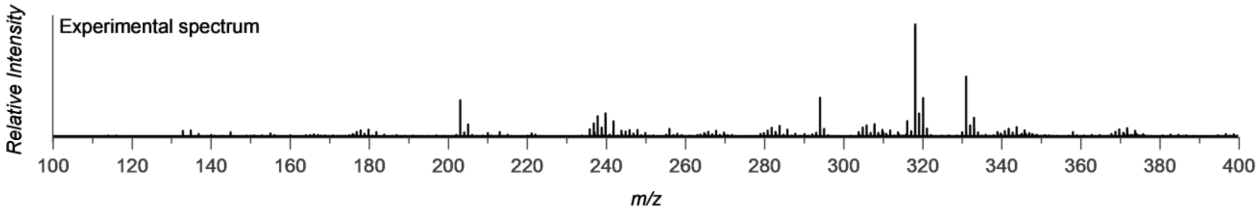

Figure S3: ESI-TOF spectra of the reaction mixture of $PdCl_2$(100 µM), *p*-benzoquinone (1 mM), styrene (5 mM) in acetonitrile and CD_3OD (1:1) ratio under CO atmosphere at 40 °C after 30-45 minutes, the black particles were filtered using syringe filter.

Below are the zoomed experimental spectrum sections (top) plotted against the calculated spectrum (bottom) for isotopic pattern analysis of the palladium complexes.

$[PdCl_2(D,styrene,CO)]^-$ *m/z* 310

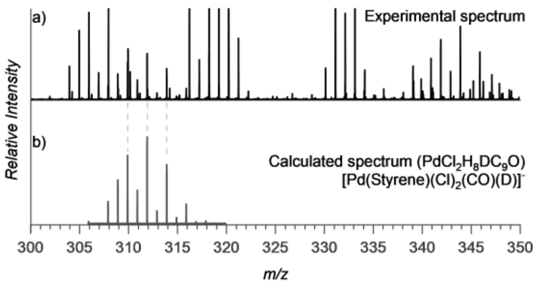

$[PdCl_2(styrene,COOCD_3)]^-$ *m/z* 342

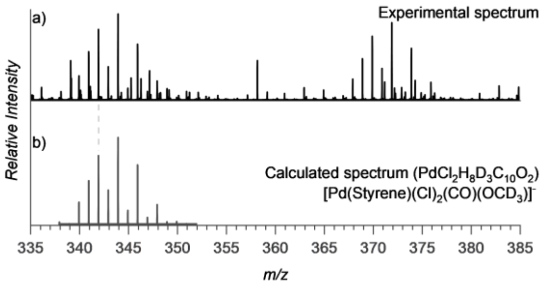

$[PdCl_2(styrene,COOCD_3,CO)]^-$ *m/z* 370

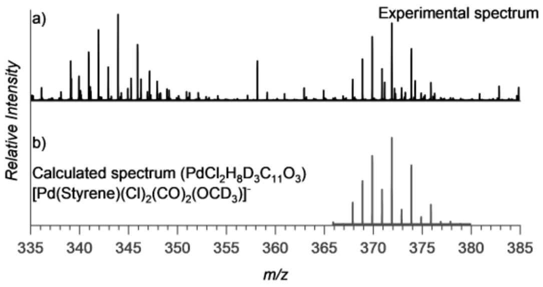

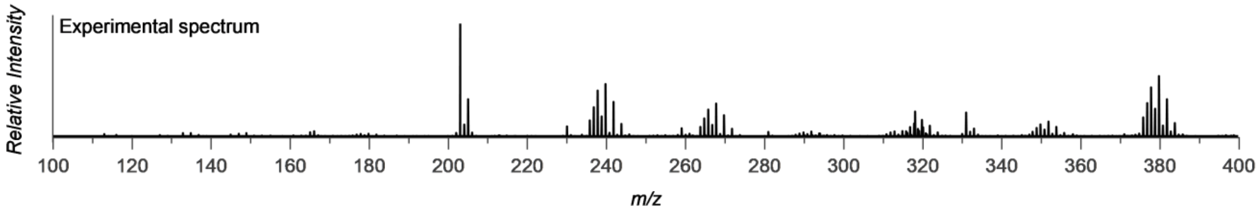

Figure S4: ESI-TOF spectra of the reaction mixture of $PdCl_2$(100 µM), *p*-benzoquinone (1 mM), D_8-styrene (5 mM) in acetonitrile and CD_3OD (1:1) ratio under CO atmosphere at 40 ℃ after 80 minutes, the black particles were filtered using syringe filter.

Below are the zoomed experimental spectrum sections (top) plotted against the calculated spectrum (bottom) for isotopic pattern analysis of the palladium complexes.

$[PdCl_2(D_8\text{-styrene},COOCD_3)]^-$ *m/z* 350

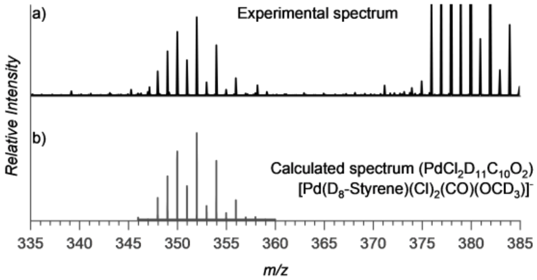

$[PdCl_2(D_8\text{-styrene},COOCD_3,CO)]^-$ *m/z* 378

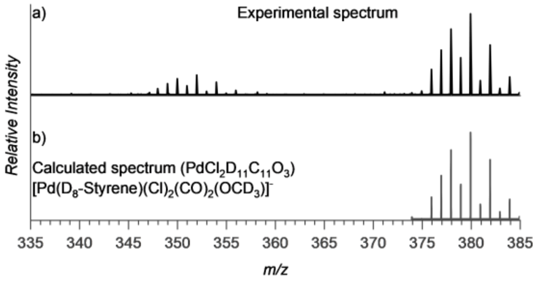

The collision induced dissociation (CID) spectra of the ions with specified *m/z*

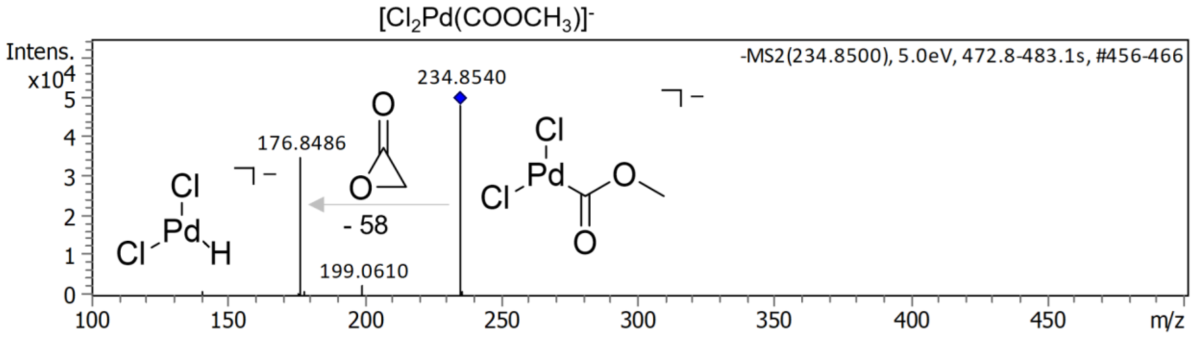

Figure S5: CID spectrum of the $[PdCl_2(COOCH_3)]^-$ with *m/z* 235 at collision energy 5 eV

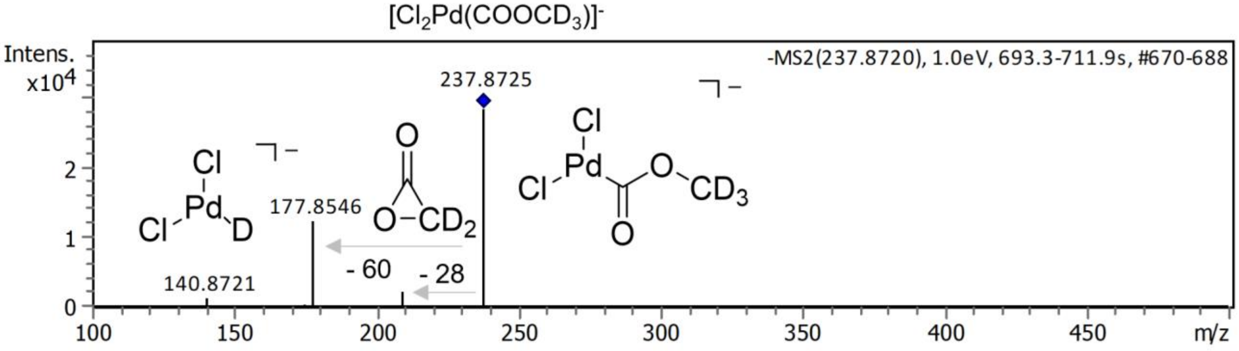

Figure S6: CID spectrum of the $[PdCl_2(COOCD_3)]^-$ with *m/z* 238 at collision energy 1 eV

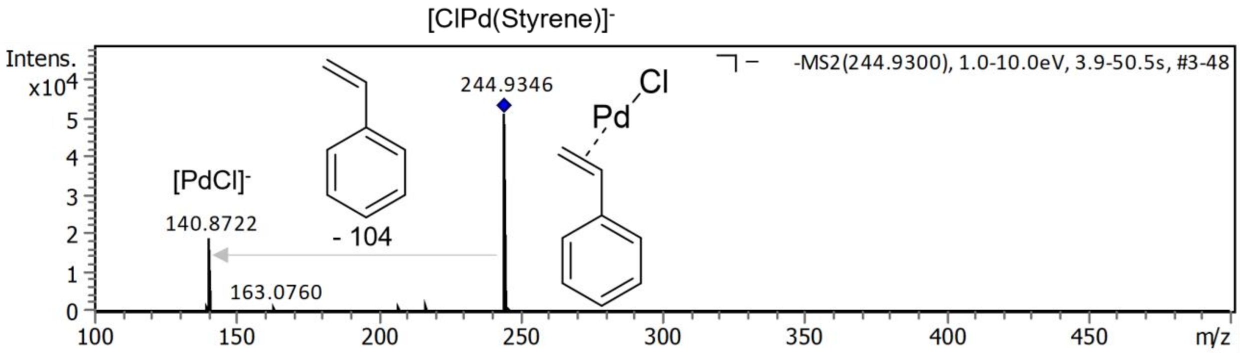

Figure S7: CID spectrum of the $[PdCl(styrene)]^-$ with *m/z* 245

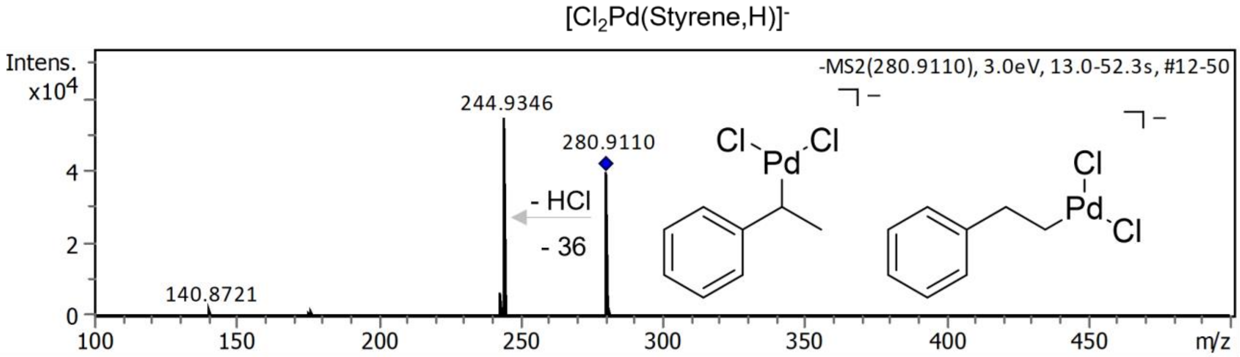

Figure S8: CID spectrum of the $[PdCl_2(H,styrene)]^-$ with *m/z* 281

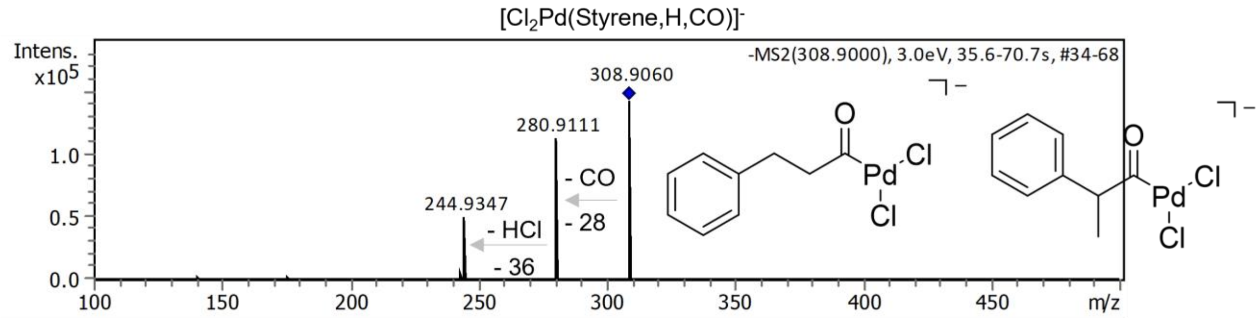

Figure S9: CID spectrum of the $[PdCl_2(H,styrene,CO)]^-$ with *m/z* 309

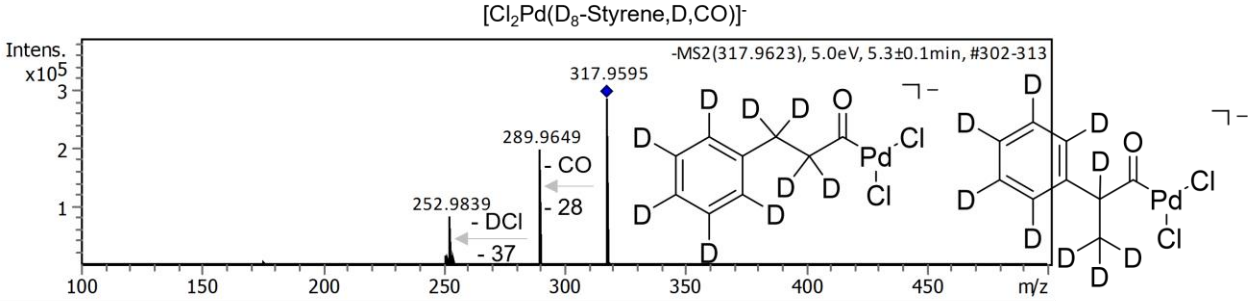

Figure S10: CID spectrum of the $[PdCl_2(D,D_8\text{-styrene},CO)]^-$ with *m/z* 318

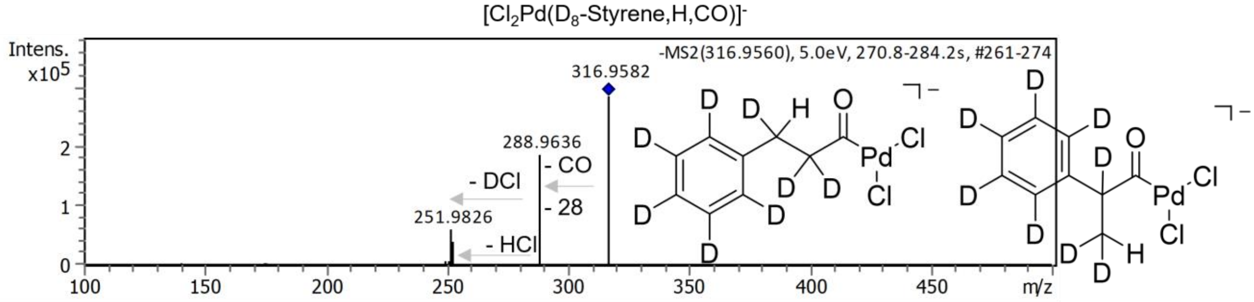

Figure S11: CID spectrum of the $[PdCl_2(H,D_8\text{-styrene},CO)]^-$ with *m/z* 317

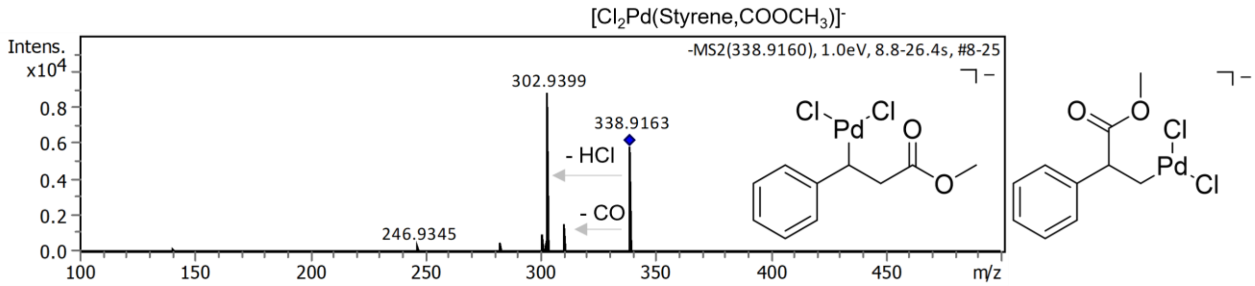

Figure S12: CID spectrum of the $[PdCl_2(styrene,COOCH_3)]^-$ with *m/z* 339

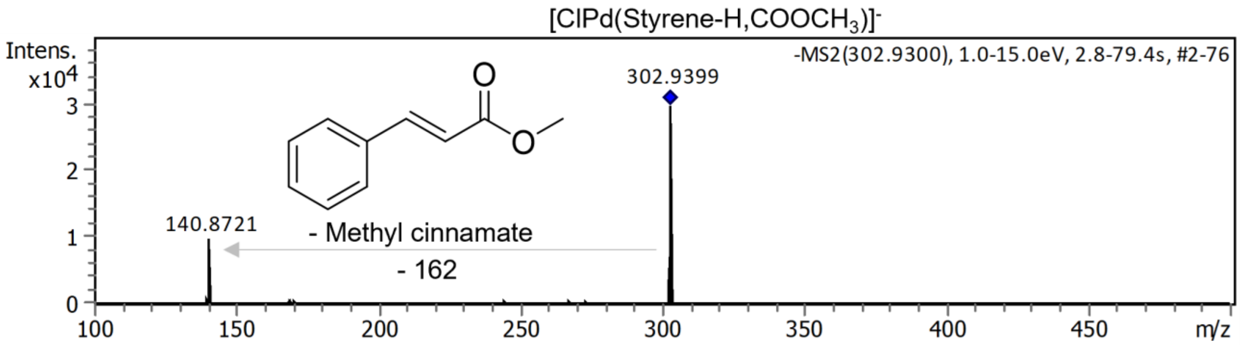

Figure S13: CID spectrum of the $[PdCl((styrene\text{-}H)COOCH_3)]^-$ with *m/z* 303

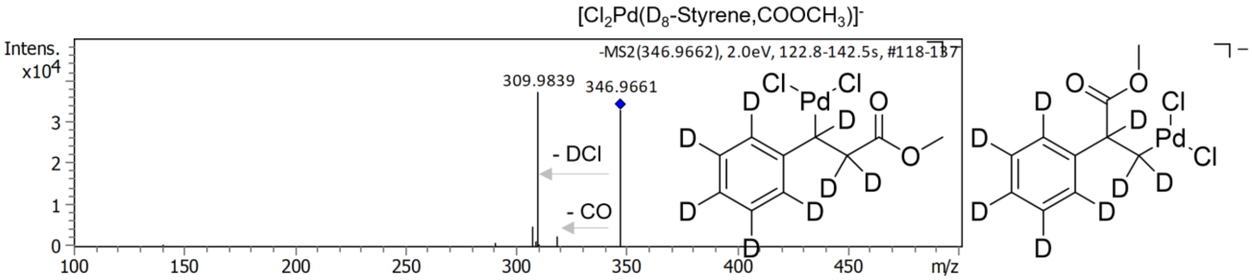

Figure S14: CID spectrum of the $[PdCl_2(D_8\text{-}styrene,COOCH_3)]^-$ with *m/z* 347

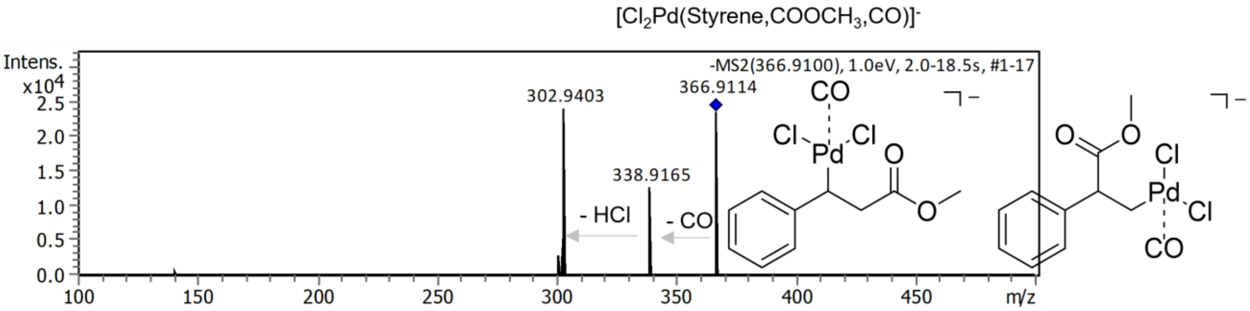

Figure S15: CID spectrum of the $[PdCl_2(styrene,COOCH_3,CO)]^-$ with *m/z* 367

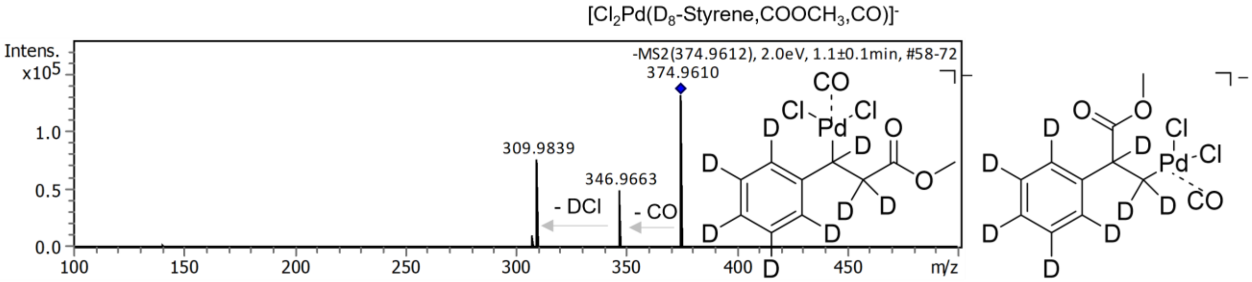

Figure S16: CID spectrum of the $PdCl_2(D_8\text{-}styrene,COOCH_3,CO)]^-$ with *m/z* 375

Energy Resolved Collision Induced Dissociation (CID) experiments

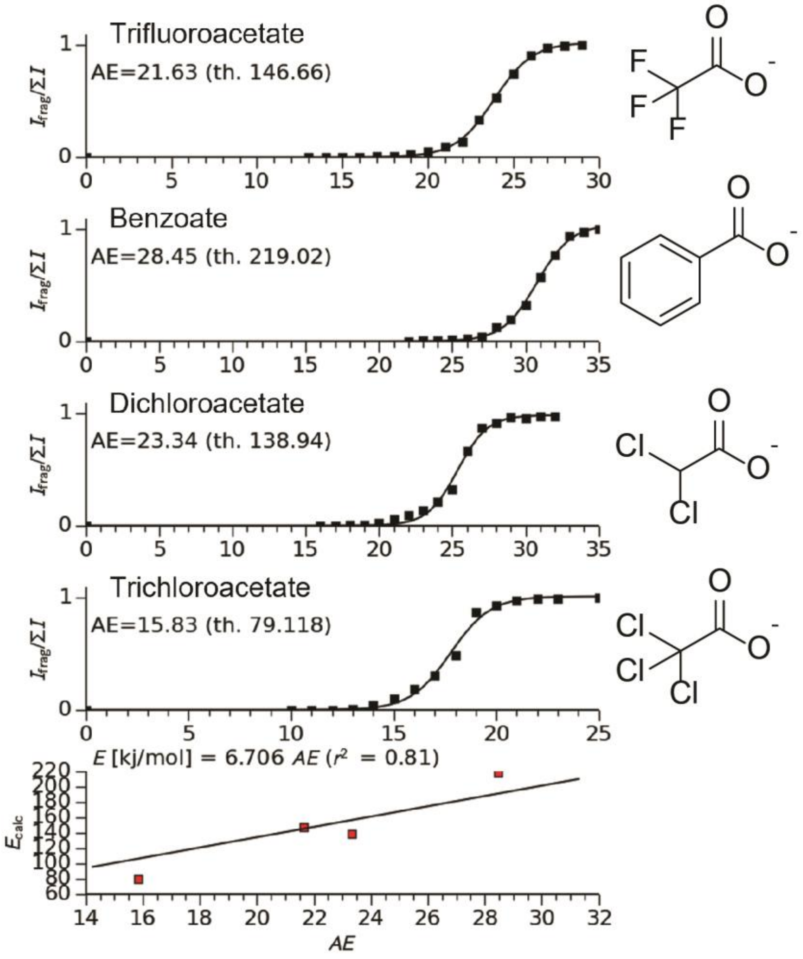

Figure S17: Calibration of the ion trap of the LCQ-Deca electrospray ionization mass spectrometer in the negative mode using specified carboxylate ions of known bond dissociation energies. The appearance energies (*AEs*) were obtained by sigmoidal fitting of the fragment intensity vs applied collision energy.

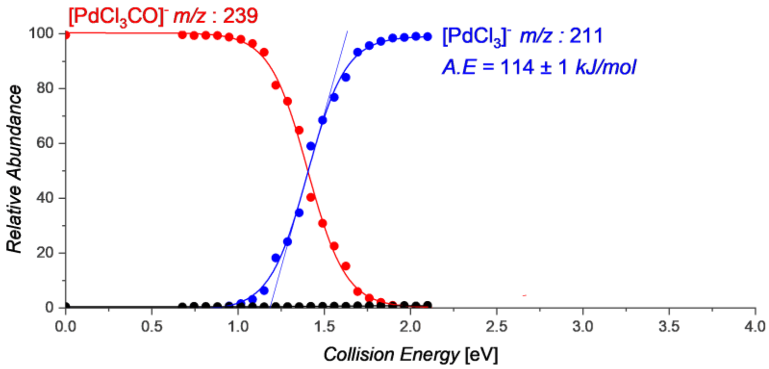

Figure S18: Energy resolved CID and extrapolation of fragmentation onset to determine the appearance energy (*AE*) of CO in the intermediate $[PdCl_3(CO)]^-$ with *m/z* 239.

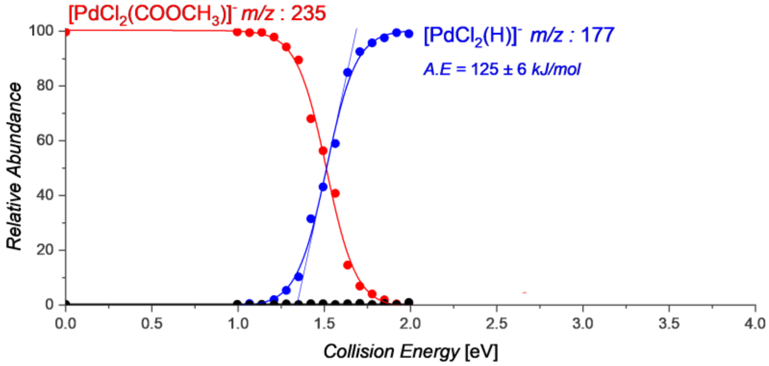

Figure S19: Energy resolved CID and extrapolation of fragmentation onset to determine the appearance energy (*AE*) of acetolactone in the intermediate $[PdCl_2(COOCH_3)]^-$ with *m/z* 235.

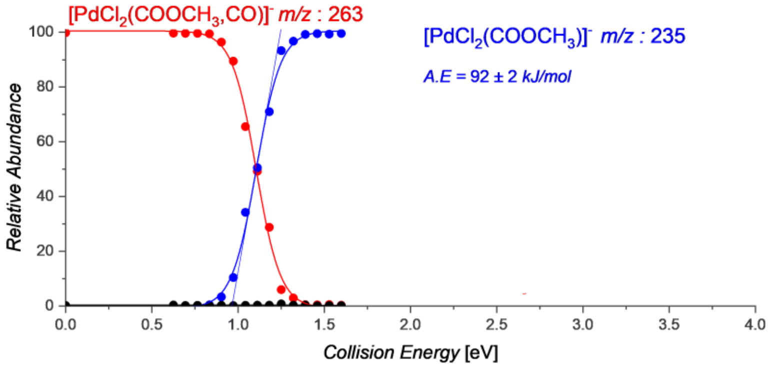

Figure S20: Energy resolved CID and extrapolation of fragmentation onset to determine the appearance energy (*AE*) of CO in the intermediate $[PdCl_2(COOCH_3,CO)]^-$ with *m/z* 263.

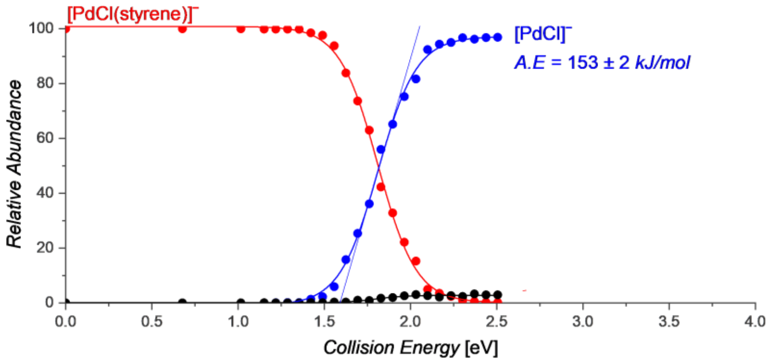

Figure S21: Energy resolved CID and extrapolation of fragmentation onset to determine the appearance energy (*AE*) of styrene in the intermediate $[PdCl(styrene)]^-$ with *m/z* 245.

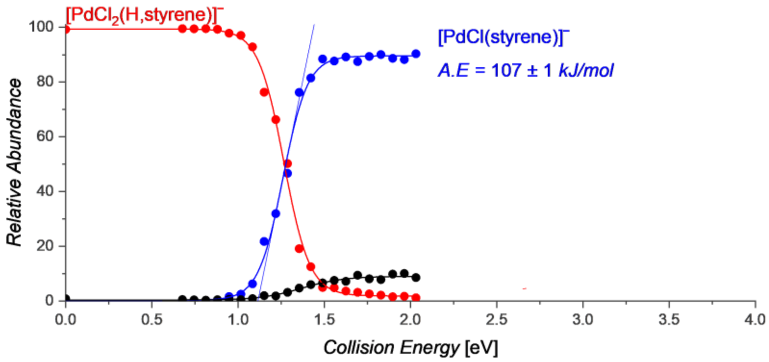

Figure S22: Energy resolved CID and extrapolation of fragmentation onset to determine the appearance energy (*AE*) of HCl in the intermediate $[PdCl_2(H,styrene)]^-$ with *m/z* 281.

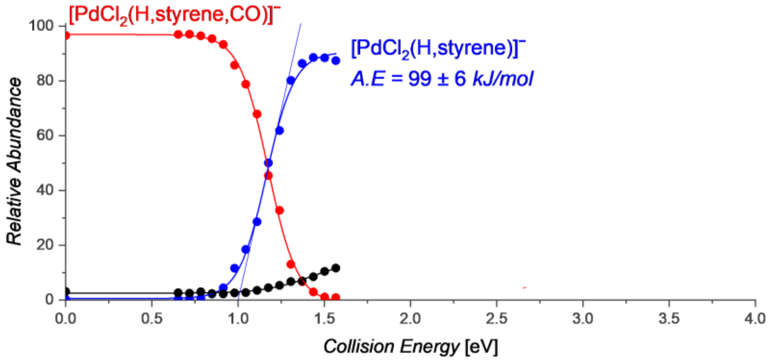

Figure S23: Energy resolved CID and extrapolation of fragmentation onset to determine the appearance energy (*AE*) of CO in the intermediate [PdCl$_2$(H,styrene,CO)]$^-$ with *m/z* 309.

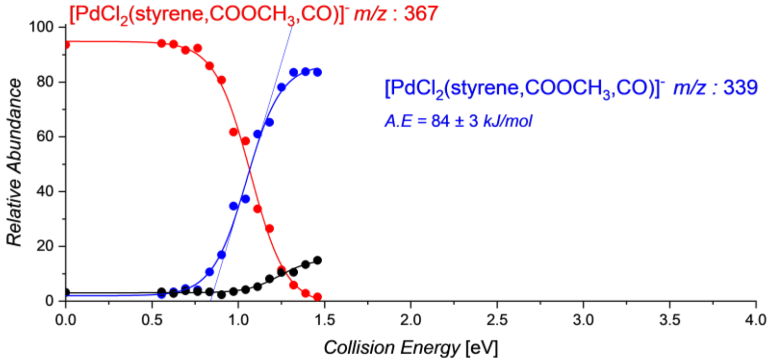

Figure S24: Energy resolved CID and extrapolation of fragmentation onset to determine the appearance energy (*AE*) of CO in the intermediate [PdCl$_2$(styrene,COOCH$_3$)]$^-$ with *m/z* 367.

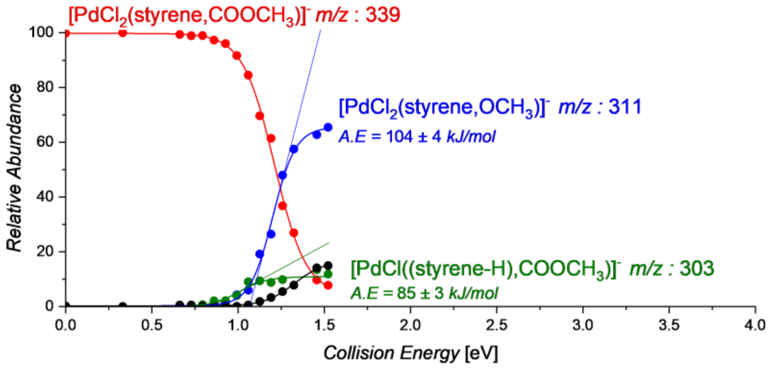

Figure S25: Energy resolved CID and extrapolation of fragmentation onset to determine the appearance energies (*AEs*) of CO and HCl in the intermediate [PdCl$_2$(styrene,COOCH$_3$)]$^-$ with *m/z* 339.

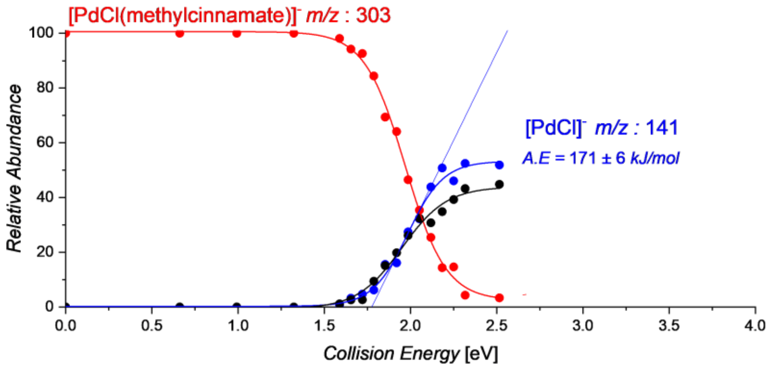

Figure S26: Energy resolved CID and extrapolation of fragmentation onset to determine the appearance energy (*AE*) of methyl cinnamate in the intermediate $[PdCl((styrene\text{-}H)COOCH_3)]^-$ with *m/z* 303.

- **Bond dissociation energy (*BDE*) calculation for CO loss from $[PdCl_2(H,styrene,CO)]^-$ *m/z* 309**

The DFT calculations were performed using the B3LYP[7] functional with the D3 dispersion correction[8] as implemented in Gaussian 16.[9] All the calculation were carried out with the 6-311+G** basis set[10,11] and SDD[12,13] on palladium. The geometries were fully optimized by verifying that there were no imaginary frequencies.

Table S1: *BDE*s calculation in kJ mol^{-1} by DFT.

$[PdCl_2(H,styrene,CO)]^-$		$[PdCl_2(H,styrene)]^-$		CO	*BDE*s
m/z 309		*m/z* 281			kJ mol^{-1}
L-Pd_2_mz309	-1472.159686	L-Pd_2_mz281	-1358.77921	-113.344011	95.7388575
B-Pd_1_mz309	-1472.159518	B-Pd_1_mz281	-1358.786661		75.735173
L-Pd_2_mz309	-1472.159686	B-Pd_1_mz281	-1358.786661		76.176257
B-Pd_1_mz309	-1472.159518	L-Pd_2_mz281	-1358.77921		95.2977735
L-Pd_2_mz309_sqpl	-1472.15256	L-Pd_2_mz281	-1358.77921		77.0295445
B-Pd_1_mz309_sqpl	-1472.157674	B-Pd_1_mz281	-1358.786661		70.893751

L-Pd_2_mz309

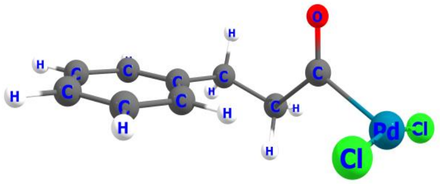

B-Pd_1_mz309

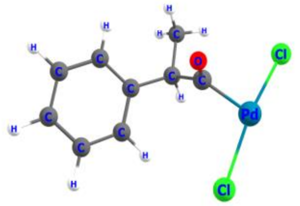

L-Pd_2_mz309_sqpl

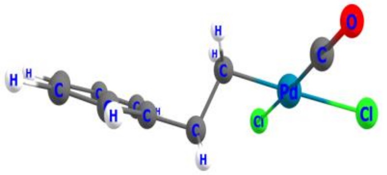

B-Pd_1_mz309_sqpl

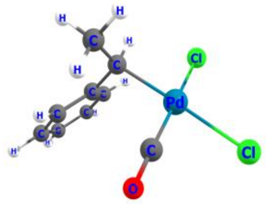

L-Pd_2_mz281

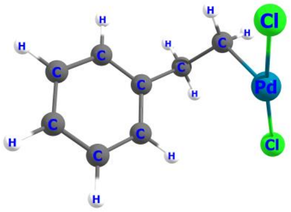

B-Pd_1_mz281

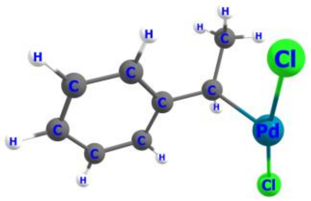

Ion mobility separation

- **Oxidant:** ***p*-benzoquinone (*p*-BQ)**

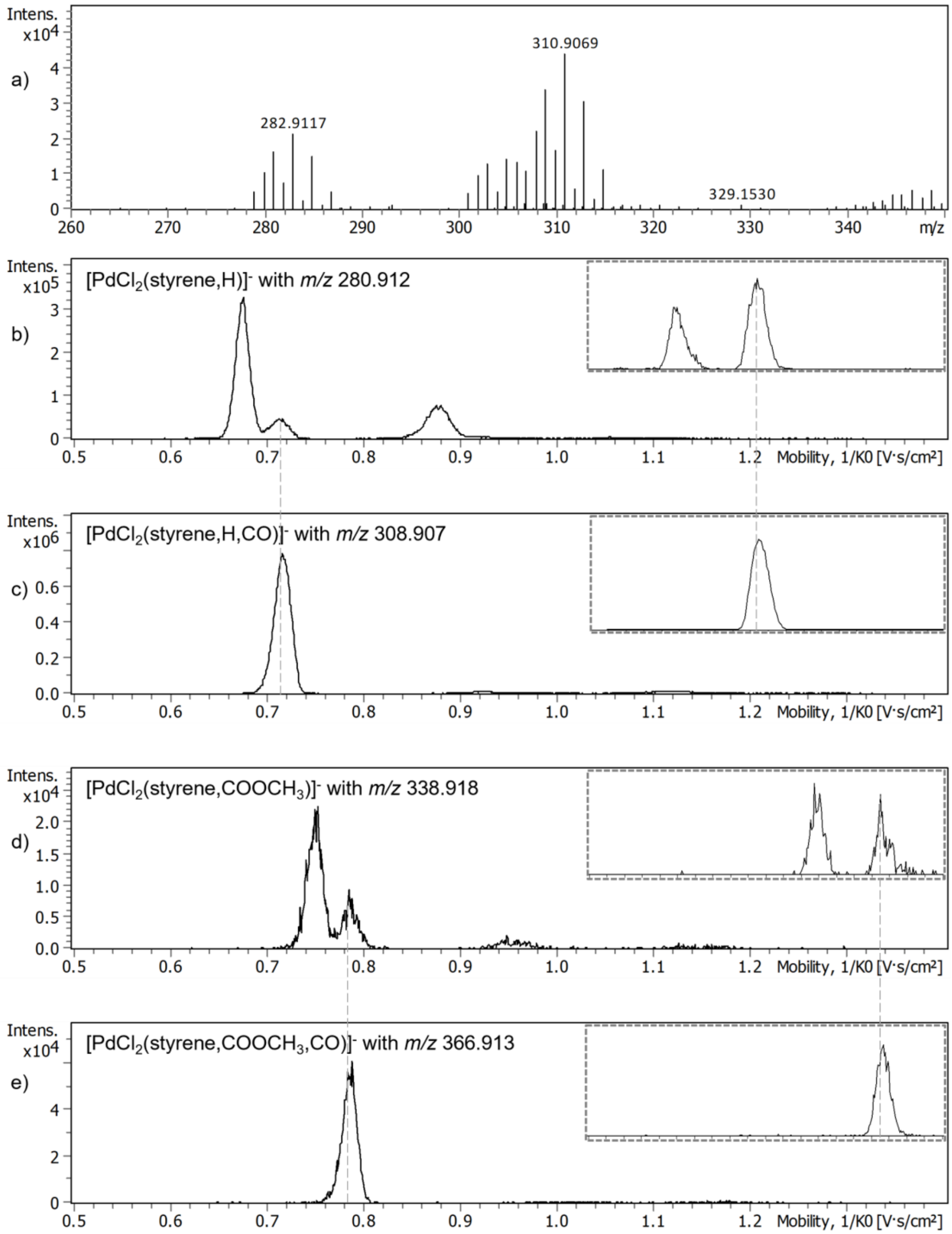

Figure S27: a) ESI-TOF spectra of the reaction mixture of $PdCl_2$ (200 µM) in a mixture of acetonitrile (1 ml) and methanol (0.5 ml), *p*-benzoquinone (1 mM), and styrene (5 mM) under the CO atmosphere at 40 ℃ for 30 min, black precipitation filtered by syringe filter; b) c) d) and e) Mobilogram of specified *m/z* with inset showing ultra resolution mobilogram from 0.65 to 0.85 range. Dashed line indicate isomer appearing due to fragmentation.

$[PdCl_2(H,styrene)]^-$ (*m/z* 281, Figure S27 b): the peak with the $1/K_0$ ~0.704 is most likely due to the fragmentation of the ions with *m/z* 309 with the same $1/K_0$; hence, the detected ions with *m/z* 281 are most likely only one isomer. We note that ultra resolution of mobility conditions are more harsh and cause fragmentation of isomers. Peak at ~ 0.88 is due to higher clusters.

$[PdCl_2(H,styrene,CO)]^-$ (*m/z* 309, Figure S27 c): one isomer only.

$[PdCl_2(styrene,COOCH_3)]^-$ (*m/z* 339, Figure S27 d): the peak with the $1/K_0$ ~0.786 is most likely due to the fragmentation of the ions with *m/z* 367 with the same $1/K_0$; hence, the detected ions with *m/z* 339 are most likely only one isomer.

$[PdCl_2(styrene,COOCH_3)(CO)]^-$ (*m/z* 367, Figure S27 e): one isomer.

- **No oxidant, stoichiometric $PdCl_2$ with styrene**

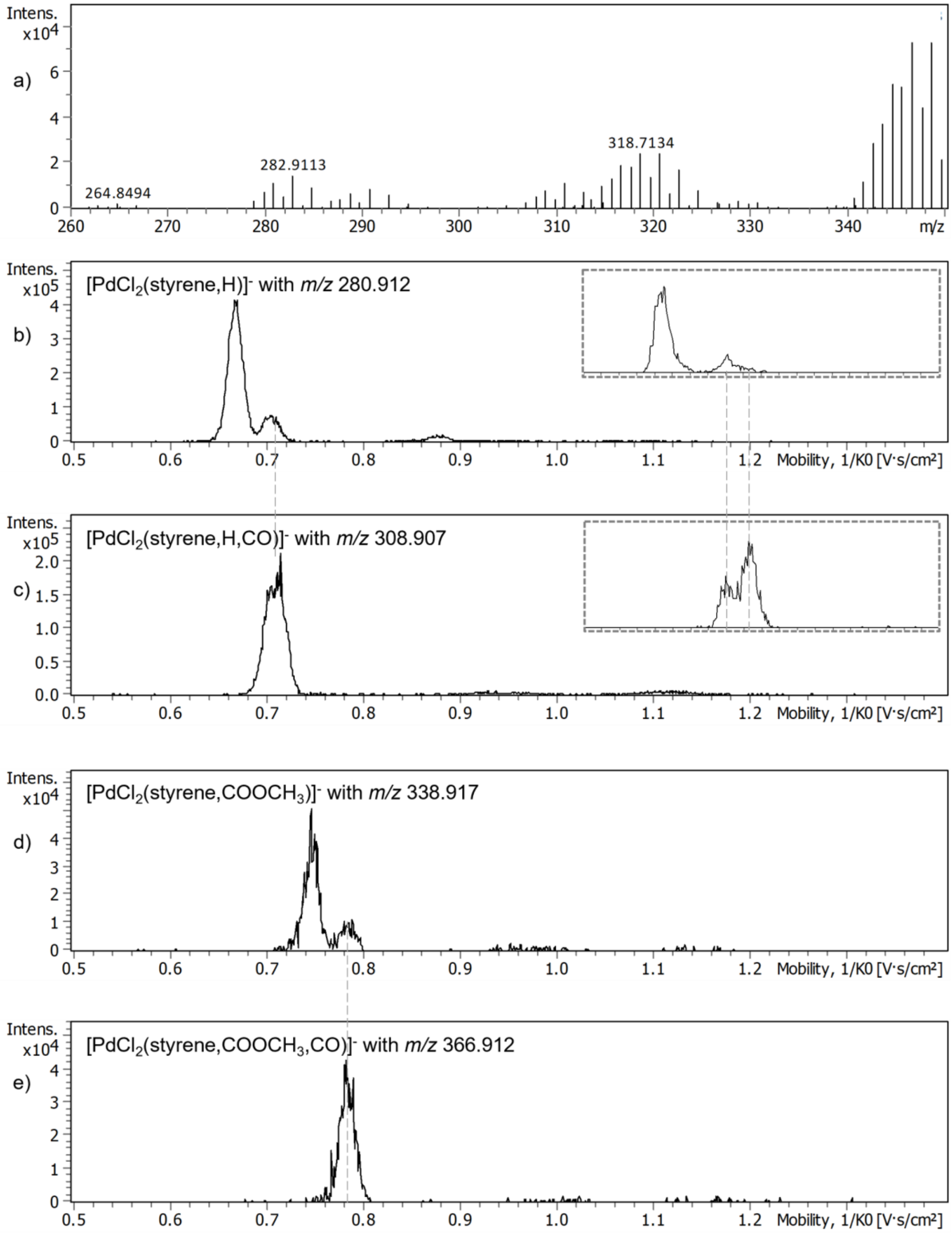

Figure S28: a) ESI-TOF spectra of the reaction mixture of excess of $PdCl_2$ and styrene in CH_3CN:MeOH under the CO atmosphere at 40 °C for 30 min, black precipitation filtered by syringe filter; b) c) d) and e) Mobilogram of specified *m/z* with inset showing ultra resolution mobilogram from 0.65 to 0.85 range. Dashed line indicates isomer appearing due to fragmentation.

$[PdCl_2(H,styrene,CO)]^-$ (*m/z* 309, Figure S28 c): Ultra resolution inset graph confirms detection of 2 isomers. When compared to Figure S27 c, new isomer with smaller reduced mobility is detected.

- **No oxidant, stoichiometric $PdCl_2$ with D_8-styrene**

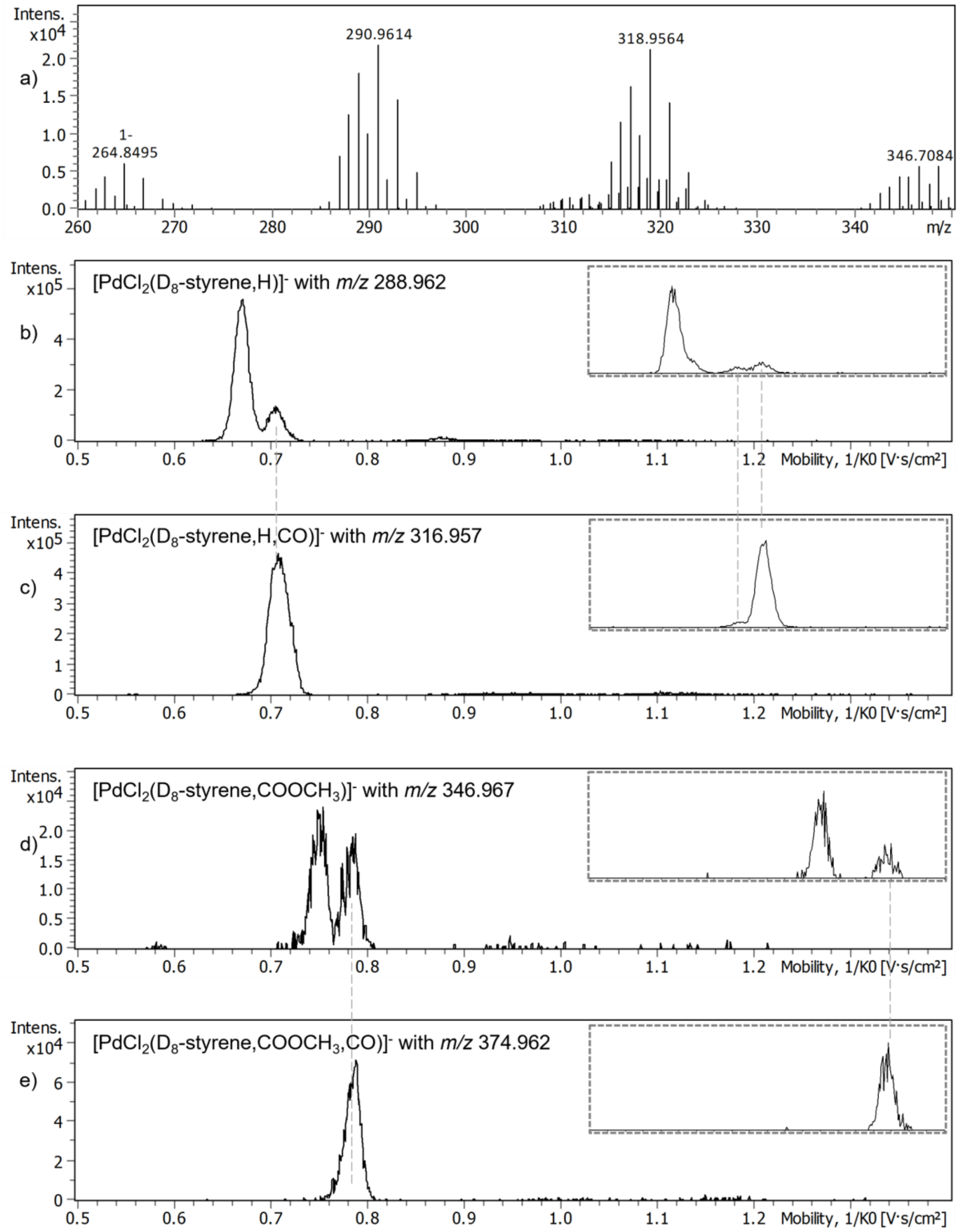

Figure S29: a) ESI-TOF spectra of the reaction mixture of excess of $PdCl_2$ and D_8-styrene in CH_3CN:MeOH under the CO atmosphere at 40 °C for 30 min, black precipitation filtered by syringe filter; b) c) d) and e) Mobilogram of specified *m/z* with inset showing ultra resolution mobilogram from 0.65 to 0.85 range. Dashed line indicates isomer appearing due to fragmentation.

$[PdCl_2(H,D_8\text{-styrene},CO)]^-$ (*m/z* 317, Figure S29 c): Ultra resolution inset graph confirms detection of 2 isomers. When compared to Figure S27 c and similar to Figure S28 c, new minor isomer with smaller reduced mobility is detected.

Collision cross section (CCS) calculation

The DFT calculations were performed using the B3LYP[7] functional with the D3 dispersion correction[8] as implemented in Gaussian 16.[9] All the calculation were done with the 6-311+G** basis set[10,11] and SDD[12,13] on palladium. The geometries were fully optimized by verifying that there were no imaginary frequencies. The CCS calculation were performed using the coordinates of optimized geometries in Collidoscope[14] 'trajectory method' based CCS calculator using N_2 as the collision gas. Ion mobility separation experiments were performed on Bruker timsTOF, the mass and mobilities were calibrated using standard calibration solution.[15] Using the DataAnalysis and Bruker Compass mobility calculator, the experimental CCS was determined.

Table S2: Experimental and calculated CCS for $[PdCl_2(H,styrene)]^-$ with *m/z* 281

	Experimental CCS (Å^2)	Calculated CCS (Å^2)	Optimized structure	Relative zero point energy difference (kJ mol^{-1})
(A) *branched type*		142.6	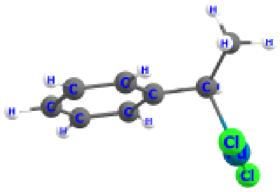	0
(B) *linear type*	140.3	145.2	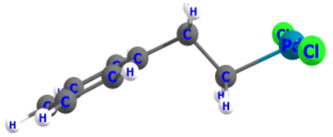	17
(C) *π type*		143.9	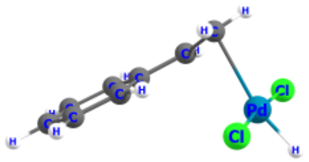	52

We note that the calculated CCS for the structure (A) and (B) is very close with only ~ 2.6 Å^2 difference, If both the isomers are present then they can be separated only based on the resolution of ion mobility separation technique used. In our case, the major isomer with ~ 140 Å^2 was detected which we assign to structure (A). It must be noted that structure (A) is 17 kJ mol^{-1} lower in energy when compared to structure (B).

Table S3: Experimental and calculated CCS for $[PdCl_2(H,styrene,CO)]^-$ with *m/z* 309

	Experimental CCS (Å^2)	Calculated CCS (Å^2)	Optimized structure	Relative zero point energy difference (kJ mol^{-1})
(A) *branched type*	148.5	150.7	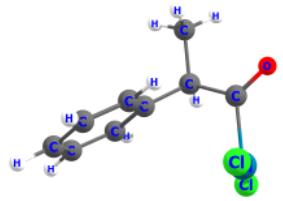	4.4
(B) *branched type'*		147.1	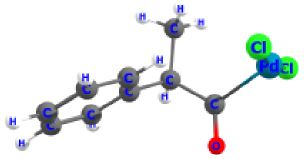	5.6
(C) *linear type*		148.7	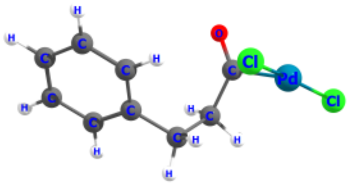	0
(D) *linear type'*		161	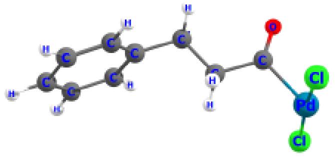	4.5
(E) *branched type and CO ligand on Pd*		152.1	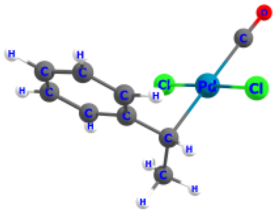	9.2
(F) *linear type and CO ligand on Pd*		154.3	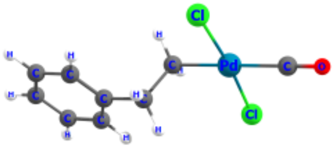	25.3

Based on the comparison of experimental and calculated CCS along with the relative energies the major isomer detected is assigned to structure (C). Experiments with no oxidant in the ultra resolution also showed a minor isomer with comparatively small CCS, which could be structure (B).

Table S4: Experimental and calculated CCS for $[PdCl_2(styrene,COOCH_3)]^-$ with *m/z* 339

	Experimental CCS (Å^2)	Calculated CCS (Å^2)	Optimized structure	Relative zero point energy difference (kJ mol^{-1})
(A) *branched ester type*		158.6	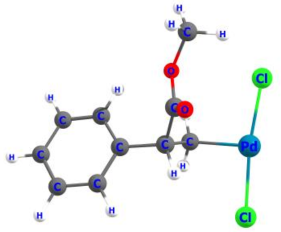	17
(B) *linear ester type*	155.1	164.9	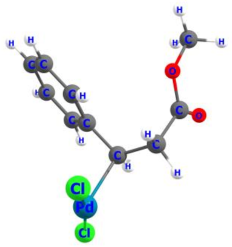	0
(C) *linear ester type'*		156.3	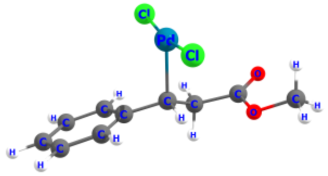	1.1

Here we note that the lowest energy structure has huge difference in CCS with the experimentally obtained CCS. Based on the comparison of experimental and calculated CCS along with the relative energies, the major isomer detected is assigned structure (C).

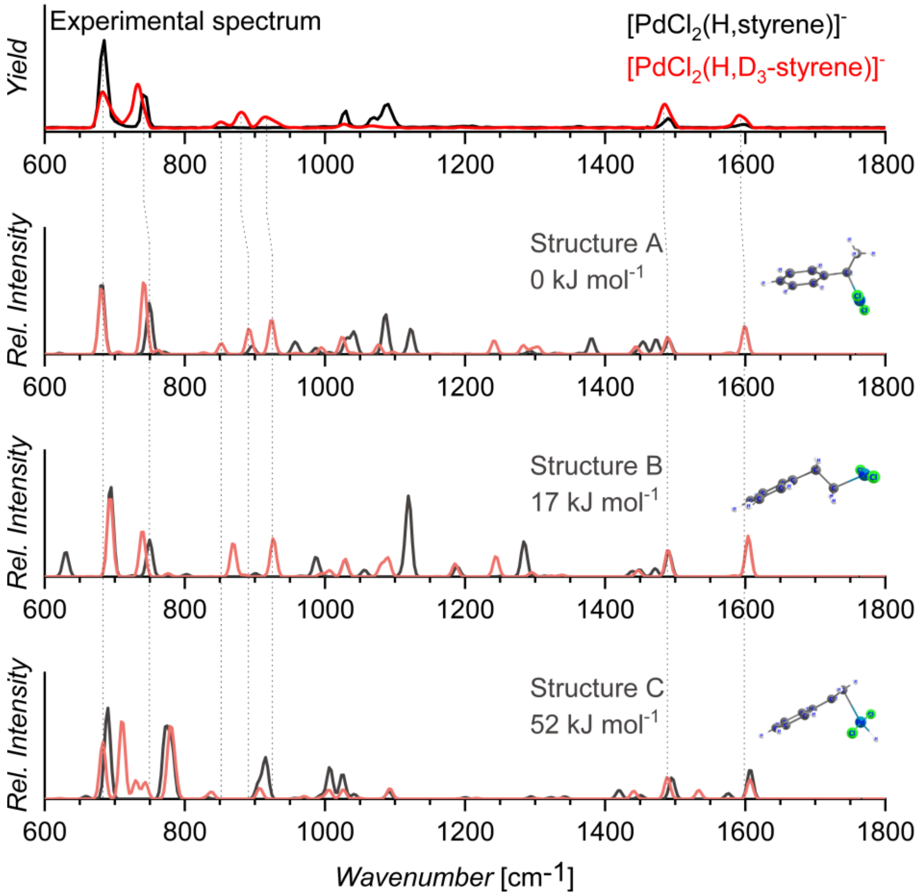

Figure S30: IRMPD of the mass selected $[PdCl_2(H,styrene)]^-$ (top experimental spectrum; black line represents *m/z* 281 for styrene while the red line represents corresponding complex with D_3-styrene) with the theoretical spectra of possible isomers (styrene-grey and D_3-styrene-light red).

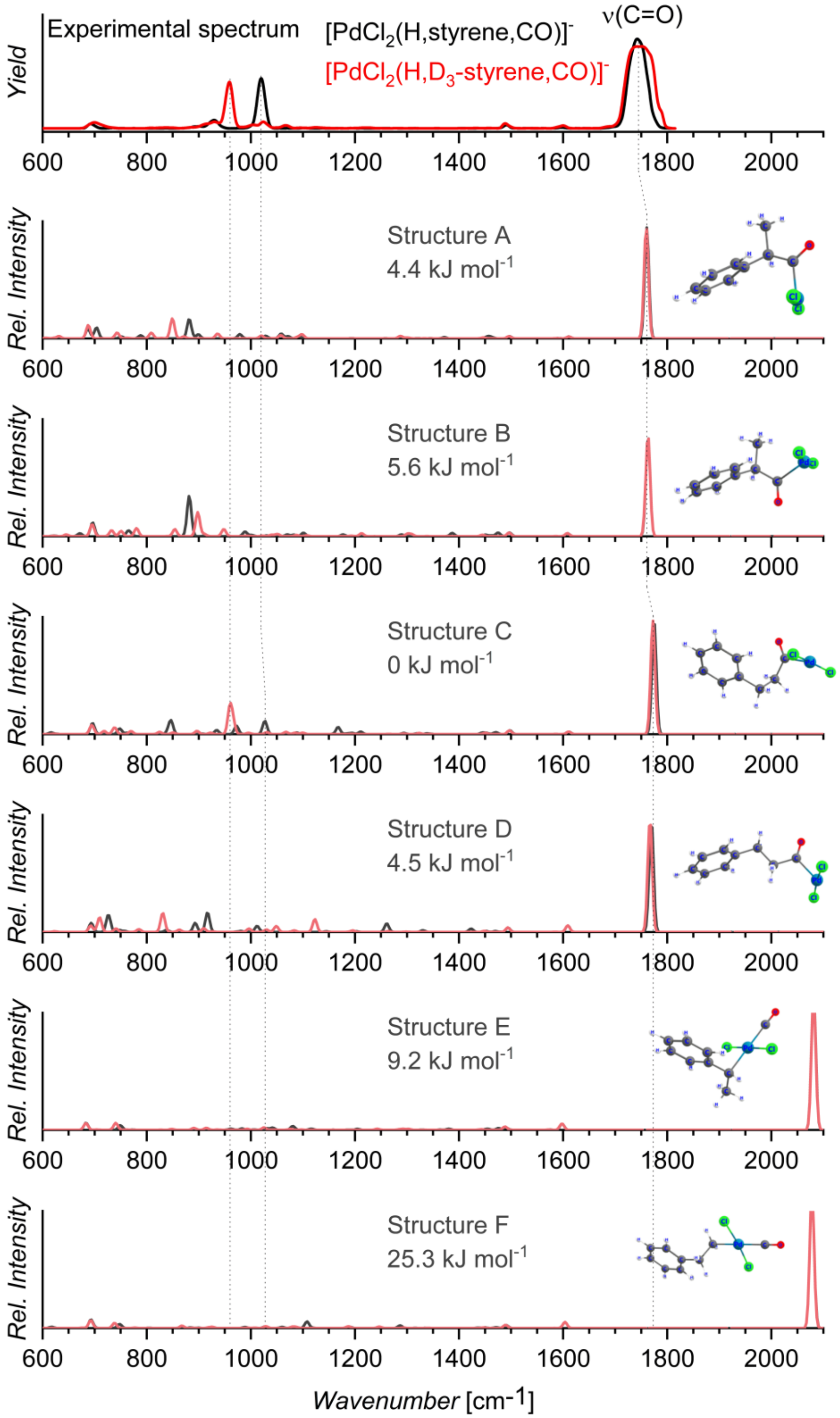

Figure S31: IRMPD of the mass selected $[PdCl_2(H,styrene,CO)]^-$ (top experimental spectrum; black line represents *m/z* 309 for styrene while the red line represents corresponding complex with D_3-styrene) with the theoretical spectra of possible isomers (styrene-grey and D_3-styrene-light red).

Delayed reactant labeling experiments

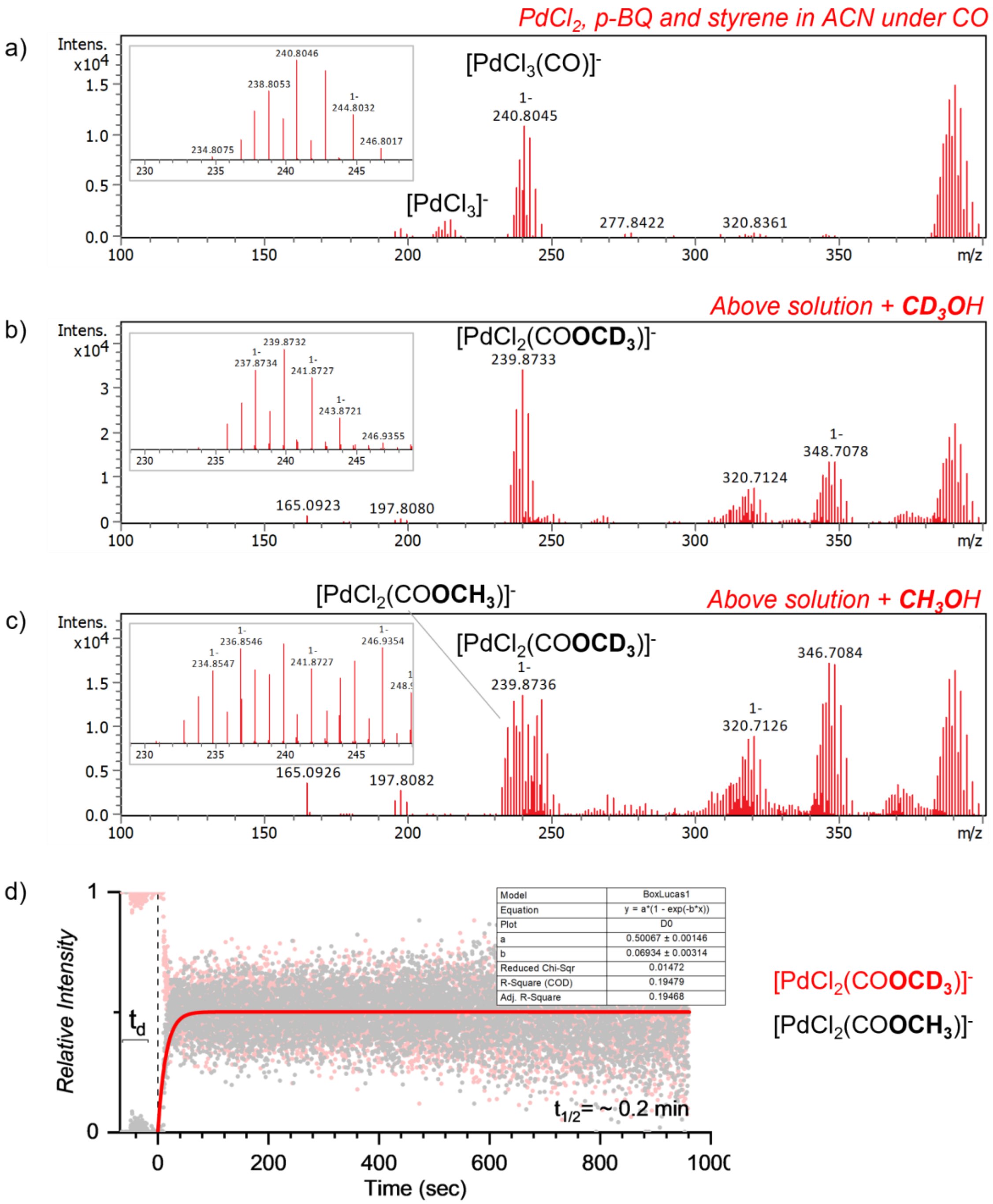

Figure S32: a) ESI-TOF spectra of the reaction mixture of $PdCl_2$(100 µM), *p*-benzoquinone (1 mM) and styrene (1 mM) in acetonitrile stirred under CO atmosphere at 40 ℃, b) CD_3OH added, c) CH_3OH added after 1 min and d) Delayed reactant labeling plot for $[PdCl_2(CO\mathbf{OCD_3})]^-$ (red points) and $[PdCl_2(CO\mathbf{OCH_3})]^-$ (black points) showing a very fast equilibration with half life time of ~ 0.2 min.

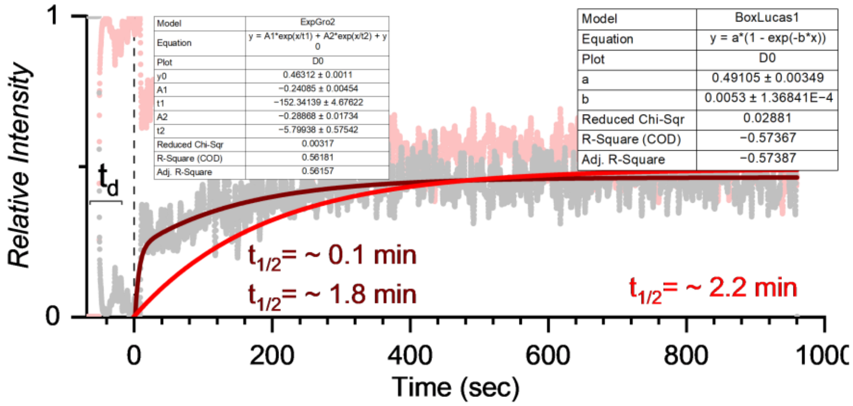

Figure S33: For the above reaction mixture, extracted delayed reactant labeling plot for $[PdCl_2(styrene,COO\mathbf{CD_3})]^-$ (red points) and $[PdCl_2(styrene,CO\mathbf{OCH_3})]^-$ (black points).

The DRL fitting to obtain $t_{1/2}$ in red did not fit the experimental data properly. Alternatively, using double exponential non linear curve fitting i.e. ExpGrow2 (dark red), gave 2 processes. As in this experiment the alcohol is added, this alcohol will first form Pd alkoxycarbonyl precursor and then the $[PdCl_2(styrene,COO\mathbf{CH_3})]^-$ intermediate.

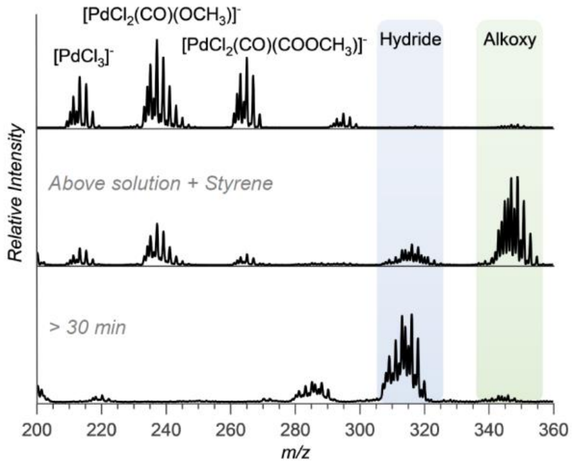

Figure S34: Snapshots of online monitoring of the reaction mixture of $PdCl_2$(100 µM), *p*-benzoquinone (500 µM) and styrene (500 µM) in acetonitrile and methanol (1:1) stirred under CO atmosphere at room temperature. Initially the alkoxy intermediates are observed, followed by hydride intermediates. At the end the intermediates of hydride cycle prevails.

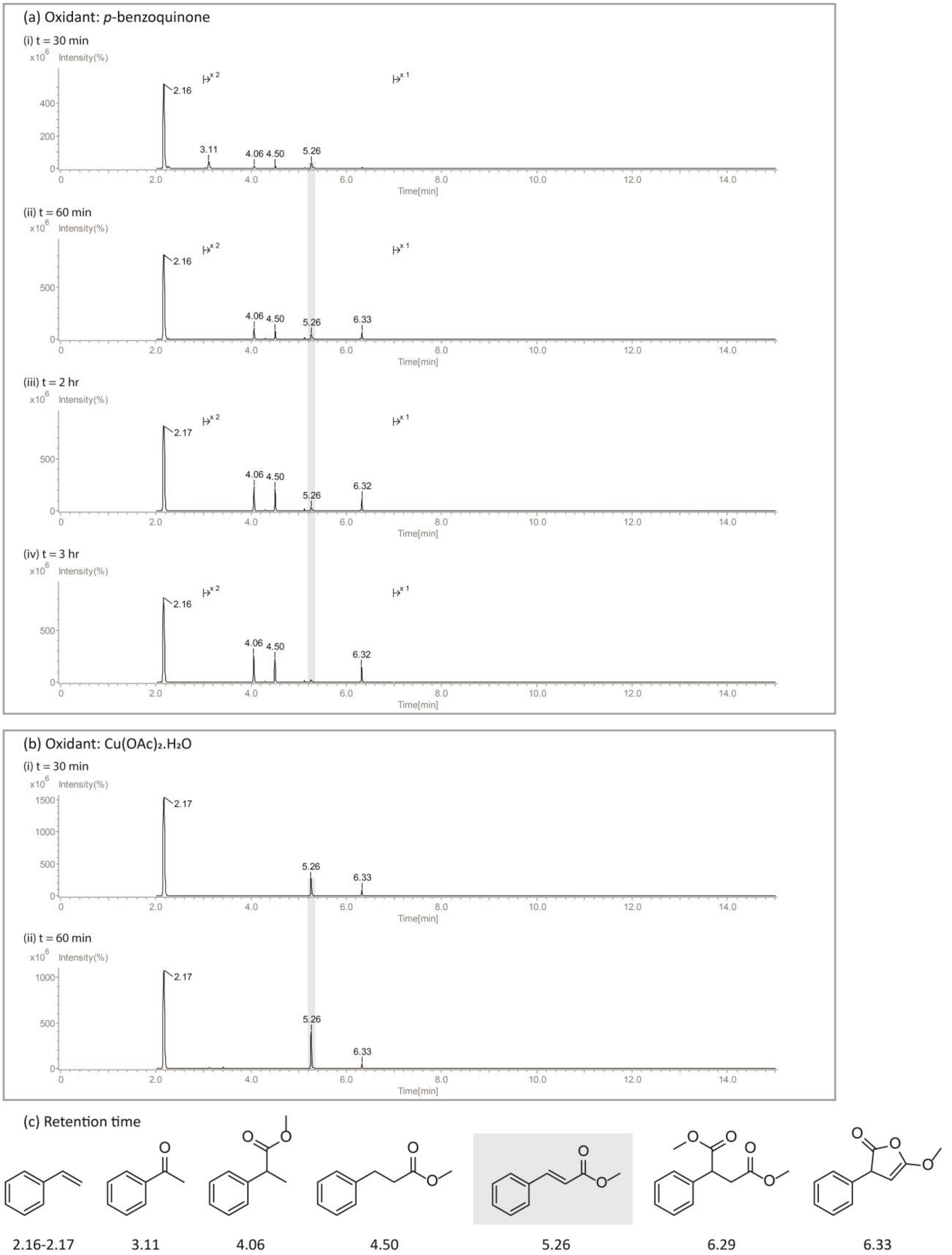

Figure S35: GC-MS chromatograms of the sample from the reaction mixture (a) $PdCl_2$ (10 mM), *p*-benzoquinone (40 mM), styrene (40 mM) in acetonitrile and CH_3OH (1:1) ratio under CO atmosphere at 40 ℃ after i) 30 minutes, ii) 60 min, iii) 2 hr and iv) 4 hr; (b) $PdCl_2$ (10 mM), $Cu(OAc)_2.H_2O$ (40 mM), styrene (40 mM) in acetonitrile and CH_3OH (1:1) ratio under CO and O_2 atmosphere at 40 ℃ after i) 30 minutes and ii) 60 min; (c) Structure are assigned based on comparison of EI spectrum with the NIST library.[citation] Except for the '6.33' which is not available in the NIST library and is proposed based on the *m/z* and fragmentation observed as a subsequent intramolecular lactonization from the dicarbonylated product.

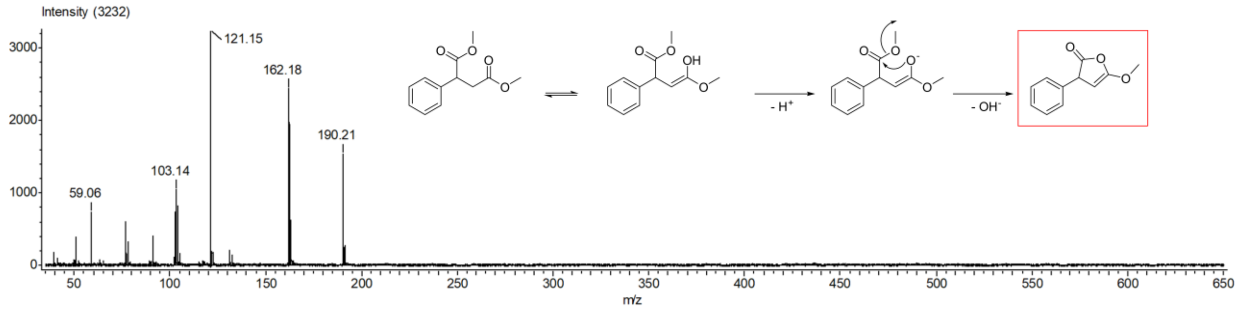

Figure S36: EI-MS spectrum of peak observed at retention time ‘6.33’ and the proposed scheme for its formation from the dicarbonylated product.

- We observe that the reaction with *p*-BQ shows unsaturated oxidative carbonylation product at ‘5.26’ at 30-60 min. When compared to other products, this unsaturated product does not grow after long time. This is in-line with our findings that the alkoxy cycle could be observed only at the start of the reaction.
- Over long time when the pH becomes slightly acidic, then the hydride cycle becomes dominant leading to the saturated products and is reflected in the chromatogram.

- Similarly, using stoichiometric $PdCl_2$ with no additional oxidant showed acidic pH and resulted in the detection of the saturated carbonylated products of the hydride cycle after 2 hr at 40 ℃.

Effect of the reaction conditions on the observation of the intermediates

- **1,1′-Bis(diphenylphosphino)ferrocene (dppf) as the bidendate phosphine ligand**

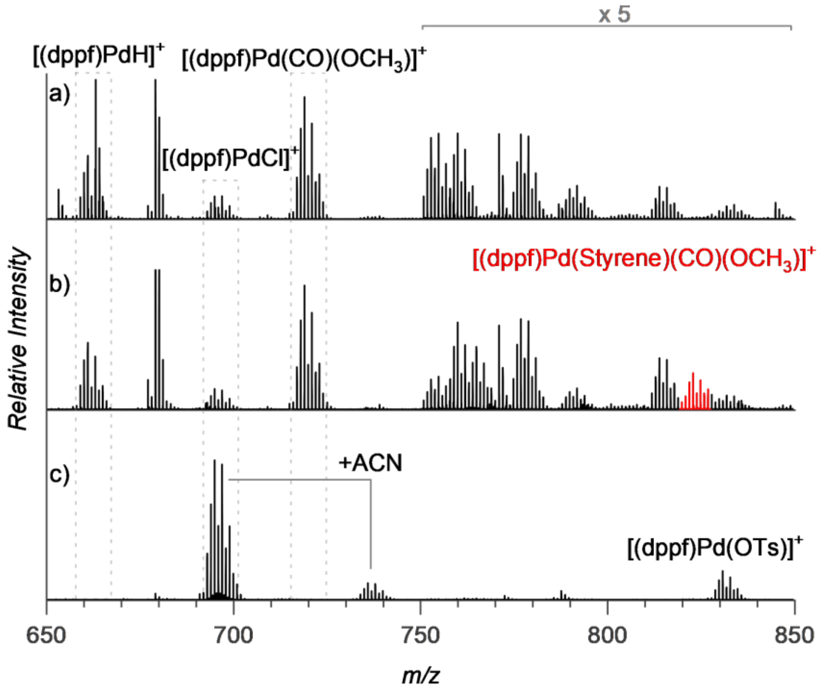

Figure S37: ESI-TOF(+) spectra, a) (dppf)$PdCl_2$, *p*-benzoquinone in CH_3CN and MeOH under CO atm, b) After styrene addition in 10min alkoxy intermediate observed and c) After acid (PTSA) addition, the alkoxy intermediate disappeared.

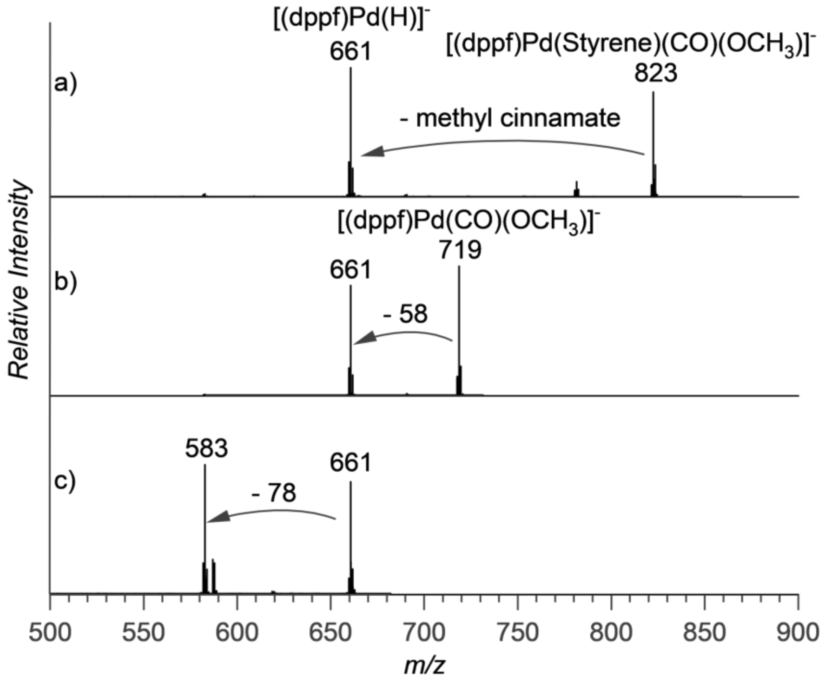

Figure S38: Collision induced dissociation (CID) spectra. a) CID of the alkoxy intermediate [(dppf)Pd(styrene,$COOCH_3$)]$^+$ ion *m/z* 823, showing the loss of the product methyl cinnamate, b) CID of the Pd alkoxy precursor complex [(dppf)Pd($COOCH_3$)]$^+$ with *m/z* 719, loss of cyclic aceto-lactone as previously observed for negatively charged precursor complexes (see Figure S5 and S6), and c) CID spectrum of [(dppf)Pd(H)]$^+$ ion with *m/z* 661.

- **Toluene/DMSO as the solvent instead of acetonitrile**

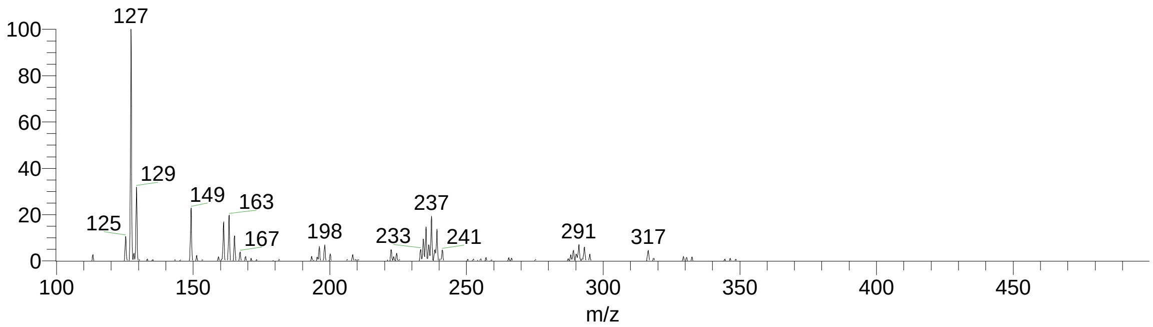

Figure S39: ESI-MS spectra of the reaction mixture of $PdCl_2$ in toluene as solvent with 5% DMSO under CO and oxygen atmosphere.
Pd-methoxycarbonyl precursor complexes $[PdCl_2(COOCH_3)]^-$ (*m/z* 235) was detected. Peak at *m/z* 289 was $[PdCl_3(DMSO)]^-$.

- **Toluene/DMSO as the solvent instead of acetonitrile and in the presence of monodentate triphenylphosphine ligand**

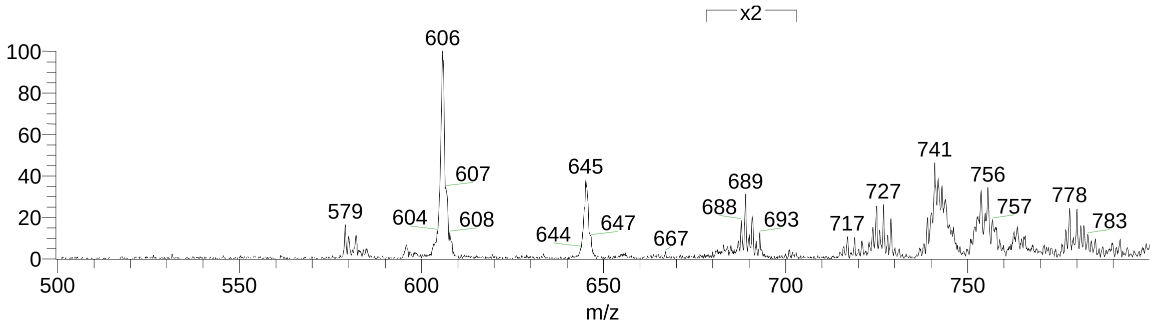

Figure S40: ESI-MS spectra of the reaction mixture of $PdCl_2(PPh_3)_2$ in toluene as solvent with 5% DMSO under CO and oxygen atmosphere.
Pd-methoxycarbonyl precursor complex with PPh_3 ligand as $[Pd(PPh_3)_2(COOCH_3)]^+$ (*m/z* 689) was detected.

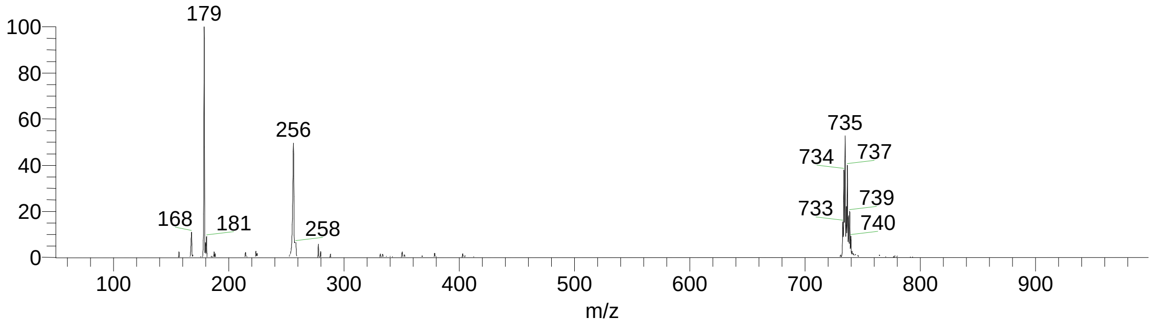

Figure S41: ESI-MS spectra of the reaction mixture of $PdCl_2(PPh_3)_2$, *p*-benzoquinone and styrene in toluene as solvent with 5% DMSO under CO and oxygen atmosphere.
Hydride cycle Pd-intermediate with PPh_3 ligand as $[Pd(PPh_3)_2(styrene,H)]^+$ (*m/z* 735) was detected.

- **Standard reaction with *p*-benzoquinone as oxidant, acetonitrile and methanol as solvent under CO, but styrene substituted to *p*-chloro styrene**

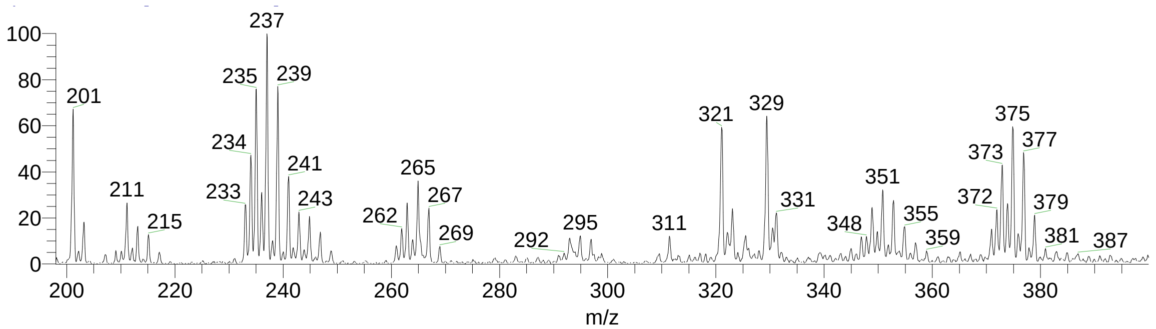

Figure S42: p-chloro styrene instead of styrene, *m/z* 373 is Pd alkoxy intermediate $[PdCl_2(Cl\text{-}styrene,COOCH_3)]^-$.

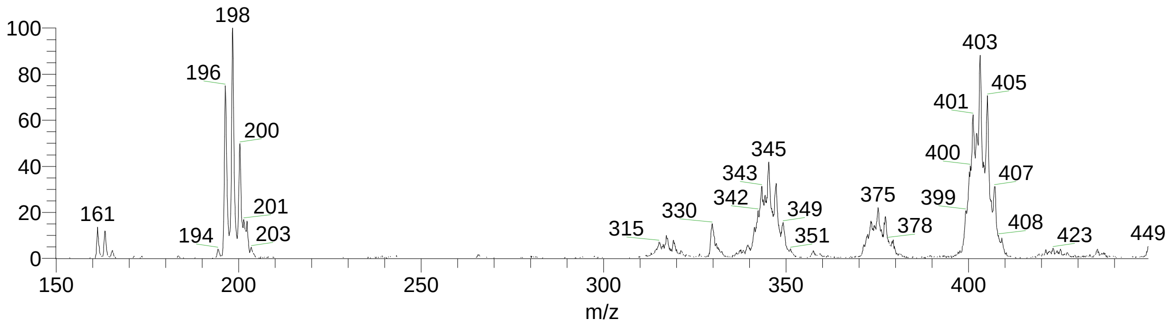

Figure S43: Chloro styrene instead of styrene after 2 hr.
Palldium hydride intermediate *m/z* 343 is Pd alkoxy intermediate $[PdCl_2(Cl\text{-}styrene,CO,H)]^-$, Palladium alkoxy intermediate $[PdCl_2(Cl\text{-}styrene,COOCH_3)]^-$ at *m/z* 375 and $[PdCl_2(Cl\text{-}styrene,COOCH_3,CO)]^-$ at *m/z* 401.

- **Copper acetate monohydrate as oxidant and in the presence of tertabutyl ammonium bromide**

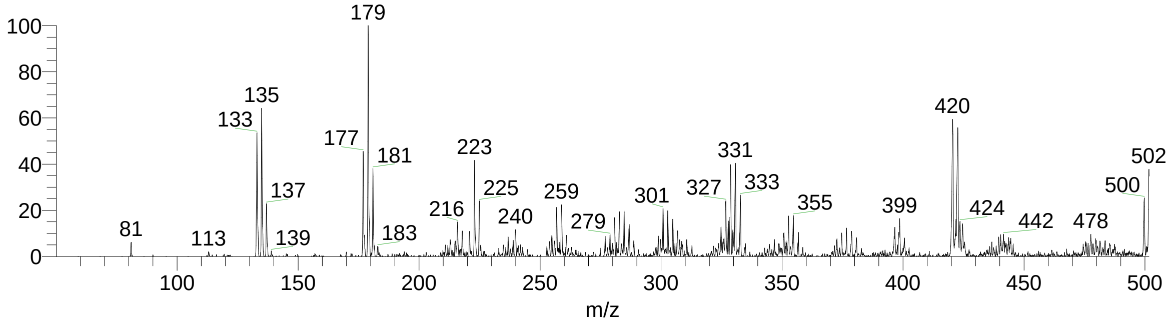

Figure S44: ESI-MS spectrum of $PdCl_2$, copper acetate monohydrate, tetrabutylammonium bromide in acetonitrile under CO atmosphere, showing various Pd and copper complex/clusters. This is pre-stirred mixture according to literature before styrene addition.[16]

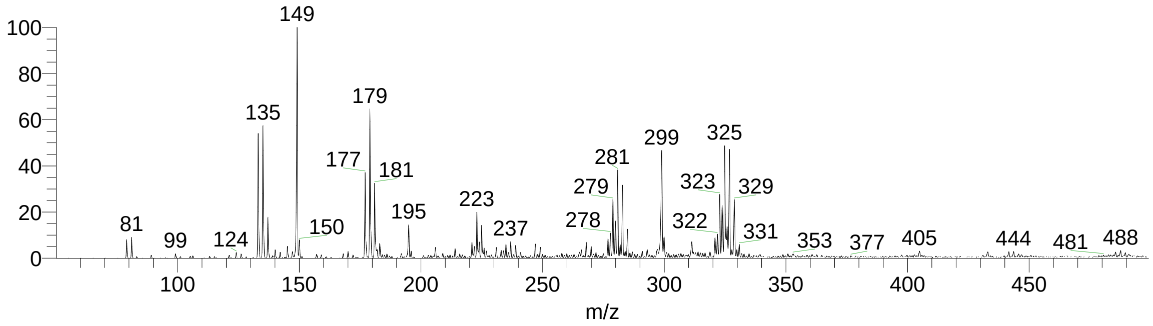

Figure S45: ESI-MS spectrum of $PdCl_2$, copper acetate monohydrate, tetrabutylammonium bromide in acetonitrile under CO atmosphere, showing various Pd and copper complex/clusters after 2 hrs. Pd alkoxy precursor complexes could be detected with bromide from TBAB exchanging with usual chloride complex. *m/z* 279 is $[PdClBr(COOCH_3)]^-$ and *m/z* 323 is $[PdBr_2(COOCH_3)]^-$.

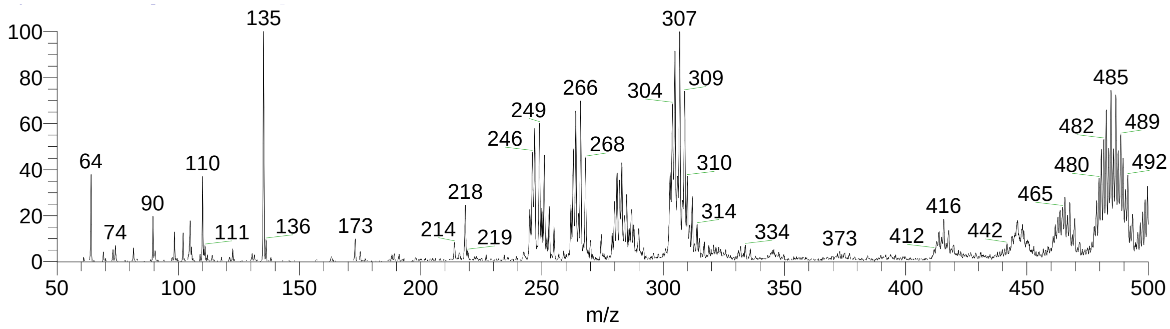

Figure S46: ESI-MS spectrum of $PdCl_2$, *p*-benzoquinone, styrene in acetonitrile:methanol under CO atmosphere, showing various Pd(II) complexes after 15 min. Pd complexes detected are as follows: *m/z* 247 $[PdCl(H)(CH_3CN)_2]Na^+$, *m/z* 264 $[PdCl(CH_3CN)_3]^+$, *m/z* 281 $[PdCl_2(CH_3CN)_2]Na^+$ and *m/z* 305 is $[PdCl(CH_3CN)_4]^+$.

It is important to note that -OMe containing complexes were not detected in this case when compared to oxidant copper acectate.

- **Palladium and copper speciation under the reaction conditions**

Stock solutions of palladium chloride 2.6 mM in CH_3CN, $Cu(OAc)_2.H_2O$ 18.7 mM in CH_3CN, styrene 144 mM in CH_3CN were prepared. The palladium chloride and copper acetate monohydrate were sonicated for 20-30 min. To 1:1 solvent mixture of CH_3CN:MeOH, 200 µM of $PdCl_2$ and 650 µM $Cu(OAc)_2.H_2O$ was added. This mixture was stirred at room temperature under the balloon of carbon monoxide (CO) and oxygen. The reaction mixture was online monitored and at 1st min excess of styrene (10 mM) was added. Resulting reaction mixture was monitored for an hour on ESI-TOF.

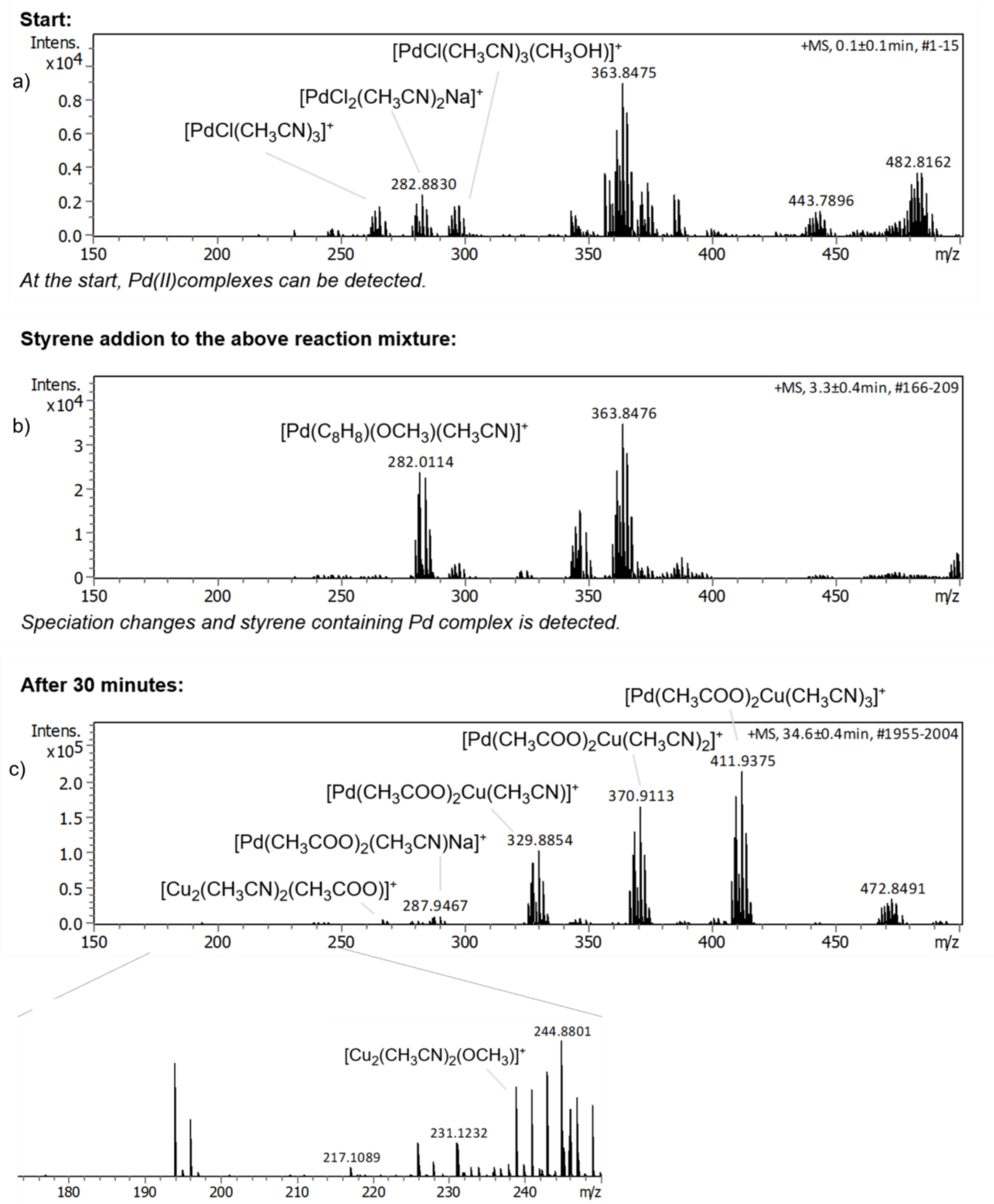

Figure S47: Snapshots of ESI-TOF online monitoring spectra of the reaction mixture of $PdCl_2$ and copper acetate monohydrate in CH_3CN:MeOH under the CO and O_2 atmosphere at room temperature a) initial spectrum, b) on addition of styrene and c) spectrum after 30 minutes, with zoomed section.

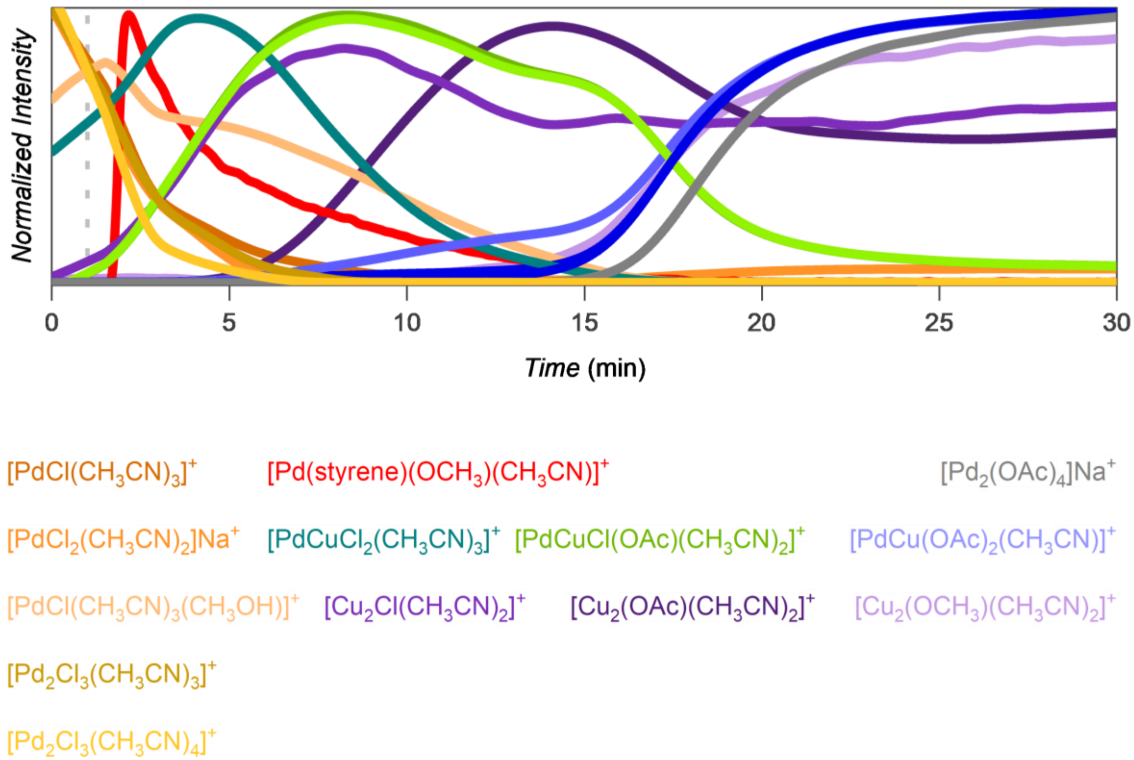

Figure S48: Plot of normalized specified ion intensities against the time.

- At the start, we observed the Pd(II) complexes as depicted in golden yellow to orange shades.
- After styrene addition (dotted grey line indicating the addition), the red curve shows that the Pd complex containing styrene increases sharply and, over time, decreases.
- The purple shades curves indicated di copper complexes with bridging ligands. It must be noted that the dicopper complexes with methoxyl as the bridging ligand are only detected after the species containing the styrene has disappeared. During this time, the Pd(II) (grey curve) is generated simultaneously.
- Bimetallic Pd Cu complexes show the interplay of the bridging ligands. Initially, complexes with chloro bridging ligands are observed (cyan), followed by mixed choro with acetato (green), and finally, towards the end, only the complexes with the acetato bridging ligands are observed (in blue).

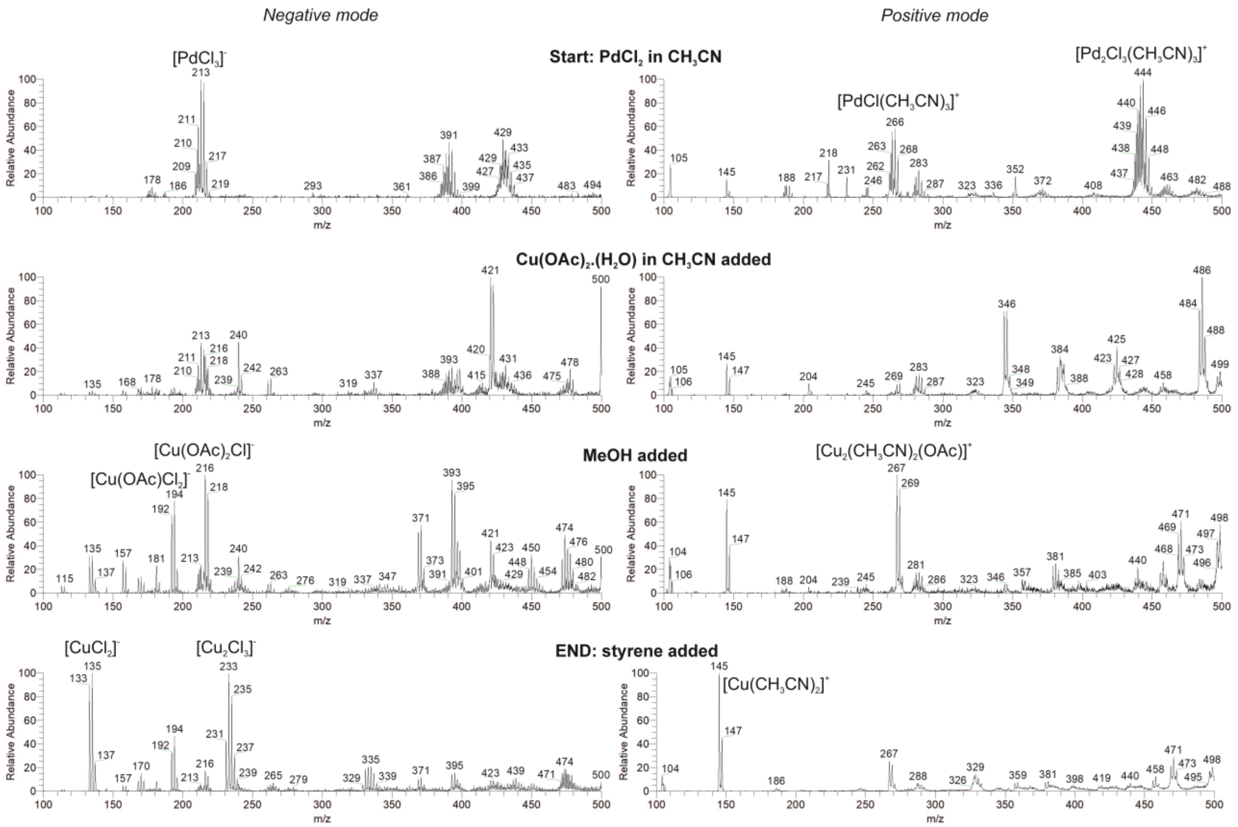

Figure S49: Plot snapshots of ESI-MS online monitoring spectra of the reaction mixture of under the O_2 atmosphere at room temperature a) initial spectrum of $PdCl_2$ in CH_3CN, b) on addition of copper acetate monohydrate, c) on the addition of methanol and d) spectrum after the addition of styrene in 20 minutes.

- At the start, we observed the Pd(II) complexes in both positive and negative modes.
- After the copper addition, followed by methanol, we initially observed copper(II) complexes in the negative mode.
- On the addition of styrene and as the reaction proceeds, copper(I) complexes are detected in both modes.

Control experiments in methanol

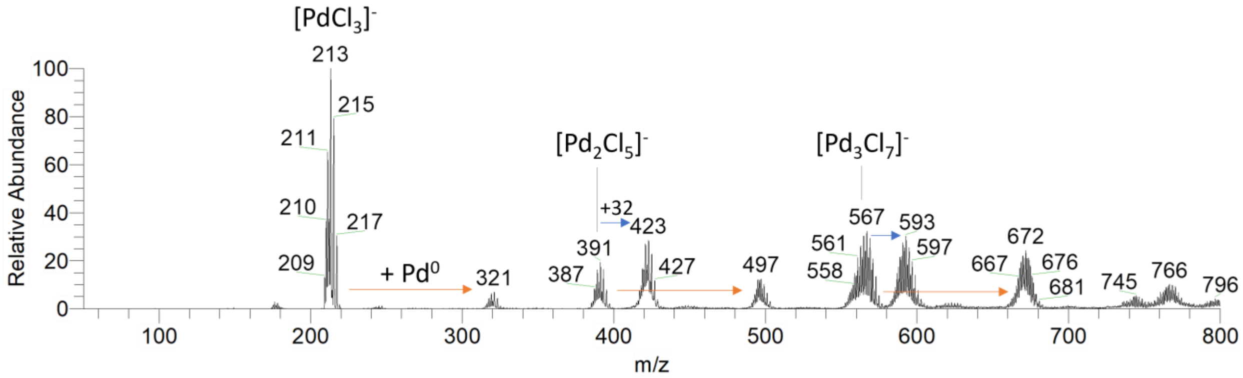

Figure S50: ESI-MS spectrum of the $PdCl_2$ in methanol (200 µM concentration, solution sonicated and filtered).

- We observed the $[(PdCl_2)_nCl]^-$ complexes and their adducts with Pd^0. Because of the low solubility of $PdCl_2$ in methanol, we had to sonicate the solution for ~20 min. During this time, some palladium was reduced. Palladium(II) complex can react with the methanol ligand by β-hydrogen elimination, leading to palladium hydride and ultimately to palladium reduction.

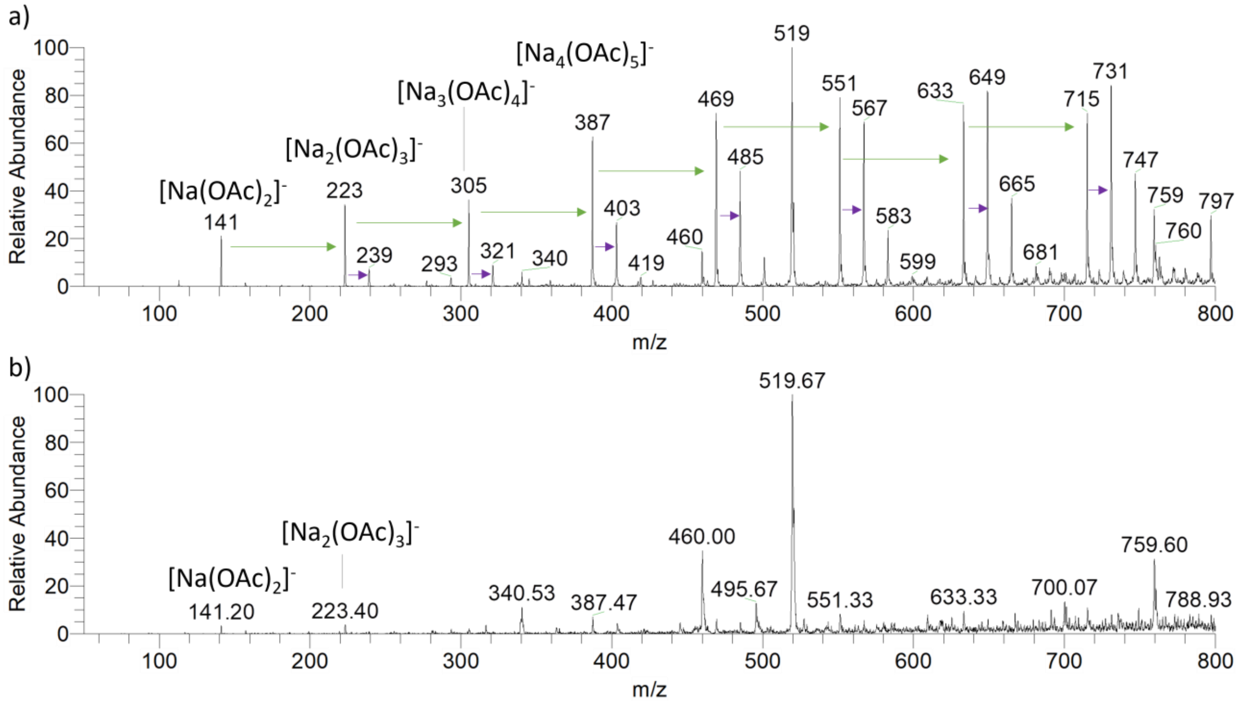

Figure S51: ESI-MS spectra a) NaOAc (200 µM) in methanol and b) $PdCl_2$ in methanol + NaOAc (both 200 µM) in methanol solutions.

- We could not detect palladium complexes from the methanol solution of $PdCl_2$ and NaOAc. Surprisingly, even the signals of the NaOAc clusters were suppressed.

xyz coordinates for optimized geometries

$[PdCl_2(H,styrene)]^-$, structure (A), zero-point energy -1358.786660

C	-3.185038000	1.481816000	-0.406891000
C	-1.939976000	1.399663000	0.208069000
C	-1.383806000	0.157377000	0.562822000
C	-2.116810000	-1.002708000	0.260382000
C	-3.362856000	-0.920086000	-0.353395000
C	-3.908442000	0.320982000	-0.687265000
C	-0.060982000	0.117613000	1.245097000
Pd	1.302825000	-0.024388000	-0.265352000
C	0.155732000	-0.964457000	2.293796000
Cl	1.074183000	-2.349434000	-0.698678000
Cl	1.836958000	2.281051000	-0.144425000
H	0.173176000	1.098565000	1.659028000
H	-1.680486000	-1.971759000	0.465302000
H	-3.905435000	-1.830870000	-0.587005000
H	-4.877909000	0.381750000	-1.171383000
H	-3.589017000	2.452840000	-0.676029000
H	-1.360480000	2.297561000	0.394701000
H	-0.572152000	-0.830425000	3.108934000
H	0.047743000	-1.969969000	1.890278000
H	1.158303000	-0.884542000	2.719424000

$[PdCl_2(H,styrene)]^-$, structure (B), zero-point energy -1358.780101

C	-4.360818000	1.204401000	-0.156766000
C	-3.016908000	1.201358000	0.213688000
C	-2.324196000	-0.000046000	0.410309000
C	-3.017067000	-1.201379000	0.213838000
C	-4.360980000	-1.204289000	-0.156618000
C	-5.040430000	0.000089000	-0.342532000
C	-0.851507000	-0.000108000	0.761533000
C	-0.004063000	0.000021000	-0.513210000
Pd	1.951991000	-0.000003000	-0.060597000
Cl	2.072023000	-2.355141000	0.006854000
Cl	2.072082000	2.355130000	0.006973000
H	-0.169940000	0.899390000	-1.108994000
H	-0.169926000	-0.899242000	-1.109160000
H	-0.611744000	0.882953000	1.359071000
H	-0.611762000	-0.883305000	1.358876000
H	-2.489488000	-2.139993000	0.351511000
H	-4.878547000	-2.147501000	-0.300590000
H	-6.086683000	0.000141000	-0.630121000
H	-4.878260000	2.147664000	-0.300855000
H	-2.489201000	2.139916000	0.351245000

$[PdCl_2(H,styrene)]^-$, structure (C), zero-point energy -1358.767006

C	4.256057000	-0.007217000	-0.631008000
C	3.479280000	1.026612000	-0.102168000
C	2.236653000	0.762525000	0.461905000
C	1.739711000	-0.550684000	0.510778000
C	2.519207000	-1.577246000	-0.045194000
C	3.767323000	-1.312475000	-0.604069000
C	0.443459000	-0.899053000	1.115046000
C	-0.308074000	-0.128949000	1.937893000

Pd	-1.485597000	0.207063000	-0.214599000
Cl	-0.772240000	2.476836000	-0.185184000
Cl	-2.405470000	-1.981414000	-0.333887000
H	-0.018971000	0.875807000	2.220740000
H	-1.186506000	-0.544228000	2.416494000
H	0.107408000	-1.914992000	0.930751000
H	2.133333000	-2.591566000	-0.041049000
H	4.351814000	-2.122964000	-1.027399000
H	5.223435000	0.206297000	-1.073834000
H	3.835966000	2.050285000	-0.147354000
H	1.620008000	1.580241000	0.811853000
H	-2.509652000	0.632989000	-1.253536000

$[PdCl_2(H,styrene,CO)]^-$, structure (A), zero-point energy -1472.158838

Pd	-1.360393000	0.291681000	-0.411151000
C	-0.748443000	-0.514433000	1.236942000
C	0.577110000	-1.299895000	1.088708000
Cl	-1.695925000	-1.793255000	-1.505303000
Cl	-1.083240000	2.543653000	0.265230000
O	-1.372821000	-0.457516000	2.254207000
H	0.278563000	-2.164952000	0.486426000
C	1.084257000	-1.773303000	2.461743000
C	1.646989000	-0.543195000	0.327545000
C	2.329317000	-1.154761000	-0.726904000
C	3.355313000	-0.487568000	-1.394644000
C	3.706398000	0.808004000	-1.018542000
C	3.018159000	1.432497000	0.021919000
C	1.998583000	0.761512000	0.690465000
H	2.032668000	-2.148855000	-1.043721000
H	3.866937000	-0.973595000	-2.219077000
H	4.495062000	1.335159000	-1.545518000
H	3.255850000	2.454146000	0.297439000
H	1.434278000	1.274013000	1.460806000
H	1.967079000	-2.405167000	2.331055000
H	1.360464000	-0.920057000	3.085784000
H	0.309479000	-2.337813000	2.983965000

$[PdCl_2(H,styrene,CO)]^-$, structure (B), zero-point energy -1472.158386

C	2.382380000	0.757706000	0.303836000
C	2.137934000	-0.614232000	0.158173000
C	3.208367000	-1.460448000	-0.141789000
C	4.502355000	-0.956786000	-0.280264000
C	4.738650000	0.408111000	-0.128575000
C	3.672121000	1.261827000	0.158889000
C	0.737565000	-1.167547000	0.348223000
C	0.270413000	-1.052654000	1.803615000
C	-0.208285000	-0.540643000	-0.721080000
O	0.021494000	-0.623547000	-1.889300000
Pd	-1.801435000	0.341857000	-0.073100000
Cl	-3.038613000	-1.679118000	0.071251000
Cl	-0.882840000	2.522993000	0.115509000
H	3.026841000	-2.522756000	-0.271708000
H	5.321405000	-1.629932000	-0.512756000
H	5.742423000	0.804832000	-0.241021000
H	3.841608000	2.328356000	0.263131000
H	1.554522000	1.430725000	0.501867000

H	0.985300000	-1.572021000	2.450202000
H	0.218271000	-0.008095000	2.116570000
H	-0.716826000	-1.500446000	1.927341000
H	0.736215000	-2.225583000	0.062284000

[PdCl$_2$(H,styrene,CO)]$^-$, structure (C), zero-point energy -1472.160521

C	3.571842000	-1.285648000	-0.656075000
C	2.454604000	-0.442775000	-0.630233000
C	2.557361000	0.784311000	0.035956000
C	3.748802000	1.152546000	0.658287000
C	4.856532000	0.305297000	0.627083000
C	4.764823000	-0.918917000	-0.034511000
C	1.156280000	-0.866026000	-1.279582000
C	0.196032000	-1.600638000	-0.312086000
Pd	-1.921100000	0.353575000	-0.005452000
Cl	-3.417280000	-1.485093000	0.119993000
Cl	-0.751848000	2.362896000	-0.470325000
H	0.736106000	-2.371293000	0.248283000
H	-0.614885000	-2.065541000	-0.874658000
H	1.371122000	-1.545787000	-2.110343000
H	0.640539000	0.004564000	-1.692388000
H	3.505492000	-2.240582000	-1.170196000
H	5.619956000	-1.586679000	-0.067508000
H	5.782050000	0.595254000	1.113901000
H	3.807903000	2.106000000	1.172621000
H	1.697482000	1.445077000	0.065182000
C	-0.432769000	-0.657751000	0.716025000
O	-0.067626000	-0.543812000	1.846544000

[PdCl$_2$(H,styrene,CO)]$^-$, structure (D), zero-point energy -1472.158806

C	-4.880040000	-1.203984000	-0.549536000
C	-3.682079000	-1.200828000	0.162755000
C	-3.064701000	0.000347000	0.532538000
C	-3.682235000	1.201390000	0.162578000
C	-4.880195000	1.204289000	-0.549714000
C	-5.485594000	0.000086000	-0.908505000
C	-1.745311000	0.000485000	1.262466000
C	-0.557558000	0.000375000	0.281056000
C	0.790429000	0.000546000	0.995524000
O	0.931032000	0.001123000	2.182360000
Pd	2.266381000	-0.000308000	-0.251828000
Cl	2.335257000	-2.362270000	-0.441994000
Cl	2.337716000	2.361467000	-0.442859000
H	-0.579328000	-0.877207000	-0.371403000
H	-0.579371000	0.877788000	-0.371643000
H	-1.667669000	-0.875754000	1.911145000
H	-1.667716000	0.876886000	1.910928000
H	-3.213035000	-2.141385000	0.434369000
H	-5.340150000	-2.147114000	-0.825490000
H	-6.417625000	-0.000014000	-1.463420000
H	-5.340429000	2.147317000	-0.825806000
H	-3.213317000	2.142048000	0.434063000

[PdCl$_2$(H,styrene,CO)]$^-$, structure (E), zero-point energy -1472.157019

C	-2.151601000	1.419072000	-0.143539000
C	-1.774722000	0.200025000	0.455678000

C	-2.544519000	-0.937817000	0.153928000
C	-3.644299000	-0.856665000	-0.696515000
C	-4.008198000	0.359968000	-1.274753000
C	-3.248791000	1.498645000	-0.993771000
C	-0.596664000	0.161782000	1.360679000
C	-0.610080000	-0.871320000	2.480472000
Pd	1.069976000	-0.027177000	0.075475000
Cl	0.629185000	-2.385382000	-0.242097000
H	-0.419224000	1.154608000	1.772048000
H	-2.254167000	-1.896599000	0.562541000
H	-4.215297000	-1.753428000	-0.917844000
H	-4.864273000	0.419306000	-1.939319000
H	-3.511489000	2.451535000	-1.443381000
H	-1.545772000	2.297578000	0.052532000
H	-1.492204000	-0.721739000	3.122118000
H	-0.625232000	-1.896115000	2.109956000
H	0.280568000	-0.761979000	3.105211000
Cl	1.515968000	2.328555000	0.435003000
C	2.647773000	-0.160478000	-1.137830000
O	3.568401000	-0.244533000	-1.800151000

$[PdCl_2(H,styrene,CO)]^-$, structure (F), zero-point energy -1472.150868

C	3.420794000	-1.200918000	0.256519000
C	2.719200000	-0.000106000	0.424019000
C	3.420583000	1.200937000	0.257269000
C	4.778529000	1.204352000	-0.057878000
C	5.465857000	0.000338000	-0.216346000
C	4.778743000	-1.203893000	-0.058634000
C	1.234238000	-0.000323000	0.715772000
C	0.415591000	0.000487000	-0.579639000
Pd	-1.605715000	0.000028000	-0.149582000
Cl	-1.495524000	-2.404874000	-0.108017000
C	-3.562053000	-0.000526000	0.279325000
O	-4.671830000	-0.000783000	0.529791000
H	0.612596000	-0.896369000	-1.170308000
H	0.612569000	0.898061000	-1.169215000
H	0.977742000	-0.884352000	1.306944000
H	0.977742000	0.882940000	1.308103000
H	2.886936000	2.139334000	0.371958000
H	5.301327000	2.147904000	-0.180317000
H	6.523032000	0.000510000	-0.461108000
H	5.301707000	-2.147277000	-0.181665000
H	2.887312000	-2.139479000	0.370622000
Cl	-1.496966000	2.404970000	-0.108161000

$[PdCl_2(styrene,COOCH_3)]^-$, structure (A), zero-point energy -1586.689403

C	-2.292053000	-0.264776000	0.057948000
C	-3.074693000	-1.313526000	0.552898000
C	-2.880575000	0.643824000	-0.832177000
H	-2.626538000	-2.030320000	1.232586000
H	-2.285053000	1.460016000	-1.220934000
C	-4.411092000	-1.450695000	0.178382000
C	-4.215658000	0.509453000	-1.205953000
H	-4.999029000	-2.272296000	0.574574000
H	-4.654279000	1.224718000	-1.894414000
C	-4.988500000	-0.537889000	-0.702465000

H	-6.027924000	-0.641808000	-0.995834000
C	-0.821587000	-0.172185000	0.454019000
C	0.077432000	-0.399971000	-0.771229000
H	0.012516000	0.421730000	-1.483316000
C	-0.506069000	1.142672000	1.155410000
H	-0.162035000	-1.355673000	-1.238697000
O	-0.283246000	1.260999000	2.336864000
H	-0.617052000	-0.947643000	1.191542000
O	-0.510370000	2.179657000	0.293742000
C	0.010398000	3.421799000	0.789975000
H	-0.516820000	3.729370000	1.695690000
H	1.076763000	3.307296000	0.989536000
H	-0.139618000	4.141946000	-0.012669000
Pd	2.011295000	-0.543999000	-0.244681000
Cl	1.699820000	-2.738140000	0.554328000
Cl	2.616817000	1.619854000	-0.947765000

$[PdCl_2(styrene,COOCH_3)]^-$, structure (B), zero-point energy -1586.695969

C	0.697425000	0.897000000	-0.129860000
C	1.240550000	1.126926000	1.145141000
C	1.856472000	2.335900000	1.448264000
C	1.941186000	3.348723000	0.490137000
C	1.392643000	3.139564000	-0.775725000
C	0.777627000	1.928751000	-1.080228000
C	0.051881000	-0.391751000	-0.510652000
C	0.710722000	-1.685037000	0.002948000
Pd	-1.870398000	-0.256872000	0.137417000
Cl	-1.501493000	-0.836386000	2.409046000
Cl	-2.660173000	0.335131000	-2.005578000
C	2.094462000	-1.809952000	-0.593456000
O	2.354508000	-2.150388000	-1.724962000
O	3.055279000	-1.443353000	0.293305000
C	4.391006000	-1.385194000	-0.223621000
H	0.760356000	-1.708202000	1.088238000
H	-0.061478000	-0.452929000	-1.592302000
H	1.142704000	0.364655000	1.906798000
H	2.262299000	2.494587000	2.442360000
H	2.417927000	4.293257000	0.731945000
H	1.438015000	3.923209000	-1.525361000
H	0.325557000	1.774453000	-2.053848000
H	0.123208000	-2.537015000	-0.340264000
H	4.449166000	-0.673736000	-1.050239000
H	5.012571000	-1.053831000	0.606811000
H	4.714158000	-2.366675000	-0.578723000

$[PdCl_2(styrene,COOCH_3)]^-$, structure (C), zero-point energy -1586.695539

C	3.835353000	-1.239860000	0.008100000
C	4.452882000	-0.381400000	-0.903000000
C	3.664409000	0.454818000	-1.695322000
C	2.278317000	0.422125000	-1.582118000
C	1.641939000	-0.443914000	-0.675293000
C	2.449052000	-1.268757000	0.125031000
C	0.155028000	-0.456774000	-0.619616000
Pd	-0.411301000	0.927392000	0.765757000
Cl	0.311156000	-0.270549000	2.671921000
C	-0.510695000	-1.801962000	-0.324660000

C	-2.020397000	-1.749175000	-0.411072000
O	-2.424473000	-1.082593000	-1.515807000
C	-3.820976000	-0.772936000	-1.594018000
Cl	-1.293204000	2.397407000	-0.858946000
O	-2.791075000	-2.251907000	0.372133000
H	-0.258041000	-0.034635000	-1.533003000
H	1.986942000	-1.897950000	0.874181000
H	4.438643000	-1.879793000	0.644150000
H	5.534589000	-0.356376000	-0.986267000
H	4.131022000	1.136793000	-2.399168000
H	1.664874000	1.087547000	-2.180469000
H	-0.169687000	-2.530808000	-1.077754000
H	-0.260642000	-2.187465000	0.661021000
H	-3.944087000	-0.214561000	-2.520092000
H	-4.423972000	-1.684400000	-1.602534000
H	-4.110058000	-0.151961000	-0.744257000

References

(1) Motloch, P.; Jašík, J.; Roithová, J. Gold(I) and Silver(I) π-Complexes with Unsaturated Hydrocarbons. *Organometallics* **2021**, *40* (10), 1492–1502. https://doi.org/10.1021/acs.organomet.1c00143.

(2) Mehara, J.; Roithová, J. Copper(II)-TEMPO Interaction. *Isr. J. Chem.* **2023**, *63* (7–8). https://doi.org/10.1002/ijch.202300011.

(3) Surendran, A. K.; Tripodi, G. L.; Pluhařová, E.; Pereverzev, A. Y.; Bruekers, J. P. J.; Elemans, J. A. A. W.; Meijer, E. J.; Roithová, J. Host-guest Tuning of the CO 2 Reduction Activity of an Iron Porphyrin Cage. *Nat. Sci.* **2023**, *3* (1). https://doi.org/10.1002/ntls.20220019.

(4) Hanzlova, E.; Váňa, J.; Shaffer, C. J.; Roithová, J.; Martinu, T. Evidence for the Cyclic CN 2 Carbene in the Gas Phase. *Org. Lett.* **2014**, *16* (20), 5482–5485. https://doi.org/10.1021/ol5027602.

(5) Thomas, G. T.; Donnecke, S.; Chagunda, I. C.; McIndoe, J. S. Pressurized Sample Infusion. *Chemistry–Methods* **2022**, *2* (1). https://doi.org/10.1002/cmtd.202100068.

(6) Bütikofer, A.; Chen, P. Cyclopentadienone Iron Complex-Catalyzed Hydrogenation of Ketones: An Operando Spectrometric Study Using Pressurized Sample Infusion-Electrospray Ionization-Mass Spectrometry. *Organometallics* **2022**, *41* (16), 2349–2364. https://doi.org/10.1021/acs.organomet.2c00341.

(7) Becke, A. D. Density-functional Thermochemistry. III. The Role of Exact Exchange. *J. Chem. Phys.* **1993**, *98* (7), 5648–5652. https://doi.org/10.1063/1.464913.

(8) Grimme, S.; Antony, J.; Ehrlich, S.; Krieg, H. A Consistent and Accurate Ab Initio Parametrization of Density Functional Dispersion Correction (DFT-D) for the 94 Elements H-Pu. *J. Chem. Phys.* **2010**, *132* (15), 154104. https://doi.org/10.1063/1.3382344.

(9) Frisch, M. J.; Trucks, G. W.; Schlegel, H. B.; Scuseria, G. E.; Robb, M. a.; Cheeseman, J. R.; Scalmani, G.; Barone, V.; Petersson, G. a.; Nakatsuji, H.; Li, X.; Caricato, M.; Marenich, a. V.; Bloino, J.; Janesko, B. G.; Gomperts, R.; Mennucci, B.; Hratchian, H. P.; Ortiz, J. V.; Izmaylov, a. F.; Sonnenberg, J. L.; Williams; Ding, F.; Lipparini, F.; Egidi, F.; Goings, J.; Peng, B.; Petrone, A.; Henderson, T.; Ranasinghe, D.; Zakrzewski, V. G.; Gao, J.; Rega, N.; Zheng, G.; Liang, W.; Hada, M.; Ehara, M.; Toyota, K.; Fukuda, R.; Hasegawa, J.; Ishida, M.; Nakajima, T.; Honda, Y.; Kitao, O.; Nakai, H.; Vreven, T.; Throssell, K.; Montgomery Jr., J. a.; Peralta, J. E.; Ogliaro, F.; Bearpark, M. J.; Heyd, J. J.; Brothers, E. N.; Kudin, K. N.; Staroverov, V. N.; Keith, T. a.; Kobayashi, R.; Normand, J.; Raghavachari, K.; Rendell, a. P.; Burant, J. C.; Iyengar, S. S.; Tomasi, J.; Cossi, M.; Millam, J. M.; Klene, M.; Adamo, C.; Cammi, R.; Ochterski, J. W.; Martin, R. L.; Morokuma, K.; Farkas, O.; Foresman, J. B.; Fox, D. J. G16_C01. 2016, p Gaussian 16, Revision C.01, Gaussian, Inc., Wallin. https://gaussian.com/citation/.

(10) Clark, T.; Chandrasekhar, J.; Spitznagel, G. W.; Schleyer, P. V. R. Efficient Diffuse Function-Augmented Basis Sets for Anion Calculations. III. The 3-21+G Basis Set for First-Row Elements, Li-F. *J. Comput. Chem.* **1983**, *4* (3), 294–301. https://doi.org/10.1002/jcc.540040303.

(11) Krishnan, R.; Binkley, J. S.; Seeger, R.; Pople, J. A. Self-consistent Molecular Orbital Methods. XX. A Basis Set for Correlated Wave Functions. *J. Chem. Phys.* **1980**, *72* (1), 650–654. https://doi.org/10.1063/1.438955.

(12) Pritchard, B. P.; Altarawy, D.; Didier, B.; Gibson, T. D.; Windus, T. L. New Basis Set Exchange: An Open, Up-to-Date Resource for the Molecular Sciences Community. *J. Chem. Inf. Model.* **2019**, *59* (11), 4814–4820. https://doi.org/10.1021/acs.jcim.9b00725.

(13) Schuchardt, K. L.; Didier, B. T.; Elsethagen, T.; Sun, L.; Gurumoorthi, V.; Chase, J.; Li, J.; Windus, T. L.

Basis Set Exchange: A Community Database for Computational Sciences. *J. Chem. Inf. Model.* **2007**, *47* (3), 1045–1052. https://doi.org/10.1021/ci600510j.

(14) Ewing, S. A.; Donor, M. T.; Wilson, J. W.; Prell, J. S. Collidoscope: An Improved Tool for Computing Collisional Cross-Sections with the Trajectory Method. *J. Am. Soc. Mass Spectrom.* **2017**, *28* (4), 587–596. https://doi.org/10.1007/s13361-017-1594-2.

(15) Chen, X.; Duez, Q.; Tripodi, G. L.; Gilissen, P. J.; Piperoudis, D.; Tinnemans, P.; Elemans, J. A. A. W.; Roithová, J.; Nolte, R. J. M. Mechanistic Studies on the Epoxidation of Alkenes by Macrocyclic Manganese Porphyrin Catalysts. *European J. Org. Chem.* **2022**, *2022* (35). https://doi.org/10.1002/ejoc.202200280.

(16) Malkov, A. V.; Derrien, N.; Barłóg, M.; Kočovský, P. Palladium-Catalyzed Alkoxycarbonylation of Terminal Alkenes To Produce α,β-Unsaturated Esters: The Key Role of Acetonitrile as a Ligand. *Chem. - A Eur. J.* **2014**, *20* (16), 4542–4547. https://doi.org/10.1002/chem.201304798.
